# Duodenal imaging on the spotlight: from A to Z

**DOI:** 10.1186/s13244-021-01045-y

**Published:** 2021-07-07

**Authors:** Carolina Terra, Daniel Ramos-Andrade, Ivo Sá-Marques, Jorge Brito, Filipe Caseiro-Alves, Luís Curvo-Semedo

**Affiliations:** 1 Department of Radiology, Centro Hospitalar e Universitário de Coimbra, Praceta Prof. Mota Pinto, 3004-561 Coimbra, Portugal; 2 Centro Hospitalar do Algarve, Faro, Portugal

**Keywords:** Duodenum, Computed tomography, Upper gastrointestinal series, Abdomen, Gastrointestinal, Magnetic resonance imaging, Ultrasound

## Abstract

Abdominal computed tomography (CT) is frequently performed to evaluate gastrointestinal pathologic conditions. The majority of the gastrointestinal radiology literature has concentrated on the colon, stomach, and distal small bowel. The duodenum is often overlooked on imaging, namely on CT, but its anatomy (intra and retroperitoneal) and location in such close proximity to other viscera results in involvement by a multitude of primary and secondary processes, some of them exclusive to this bowel segment. While some conditions, like duplications, lipomas, and diverticula, are usually asymptomatic and are incidentalomas that have no pathologic significance, others are symptomatic and very relevant and should be recognized by every general radiologist: development conditions such as annular pancreas and gut malrotation; inflammatory processes such as ulcers and secondary involvement from pancreatitis; neoplastic conditions such as adenocarcinoma, lymphoma, or local extension from adjacent malignancies. They all can be reliably diagnosed with CT. In this article, we demonstrate the typical imaging features of various diseases involving the duodenum, such as developmental, traumatic, inflammatory, infectious, neoplastic, and postsurgical pathologic conditions in alphabetical order, focusing mainly on upper gastrointestinal series (UGIS) and CT but also some radiography, ultrasound, and magnetic resonance (MR) imaging.

## Key points

- Duodenal pathology is often overlooked on imaging, namely on CT.
- Its location (intra/retroperitoneal and vicinity to other viscera) results in involvement by a multitude of primary and secondary processes.
- CT plays a major role in the diagnosis of traumatic duodenal injury and post-operative complications and helps in the diagnosis of other conditions such as infections, inflammations, and neoplams.

## Anatomy

The duodenum is a C-shaped hollow viscus located at the level of L1-3 vertebrae, that lies in front of the vertebral column, closely related to the head of the pancreas.

The duodenum is composed of four parts. It begins at the duodenal bulb as a continuation of the pylorus. The first part (D1) is anterior to the common bile duct, portal vein, and gastroduodenal artery, superior to the pancreatic head, and posterior to the gallbladder and liver. The second part (descending, D2) begins at the superior duodenal flexure and ends at the inferior duodenal flexure. It is anterior to the right kidney, ureter, and adrenal gland, and medial to the ascending colon and hepatic flexure, with the pancreatic head located medially. The major and minor duodenal papillae are situated on its medial wall. The third (horizontal, D3) part begins at the inferior duodenal flexure and passes transversely to the left, crossing the vertebral column. Posteriorly lies the right psoas muscle, aorta, and inferior vena cava and anteriorly the small bowel mesentery root. The fourth (ascending, D4) part passes superiorly and then curves anteriorly and terminates at the duodenojejunal flexure where it continues as the jejunum. The left psoas muscle and the aorta lie posteriorly, with the stomach in a superior position.

The arterial supply is provided by the right gastric and right gastroepiploic arteries in the duodenal cap (first 2.5 cm), superior pancreaticoduodenal artery in the remaining D1 to mid-D2, and inferior pancreaticoduodenal from mid-D2 to the ligament of Treitz. Venous drainage occurs through the prepyloric vein in the duodenal cap and superior and inferior pancreaticoduodenal veins in the remaining duodenum. Duodenal nodes drain distally to superior mesenteric nodes and proximally to celiac nodes. It is an intraperitoneal organ for the 2–3 first centimeters with the remaining length being retroperitoneal (anterior pararenal space).

Its location, with both intraperitoneal and retroperitoneal segments, and proximity to other organs, results in duodenal involvement by a multitude of primary and secondary processes.

In this review, we demonstrate the imaging features of several diseases involving the duodenum, in alphabetical order, to facilitate navigation through this article, focusing mainly on upper gastrointestinal series (UGIS) and computed tomography (CT) but also some radiography, ultrasound (US), and magnetic resonance (MR) imaging.

## From A to Z

### Adenocarcinoma

Adenocarcinoma is uncommon (only 0.4% of all GI tract tumors) but is the most common malignant tumor of the duodenum [1]. It is most often sporadic, although associations with Familial Adenomatous Polyposis, Peutz-Jeghers syndrome, and Crohn’s disease (CD) have been reported.

In UGIS, it may manifest as an annular constricting wall thickening or a polypoid or ulcerative mass. “Apple-core” lesions are a classical finding.

**Fig. 1:**
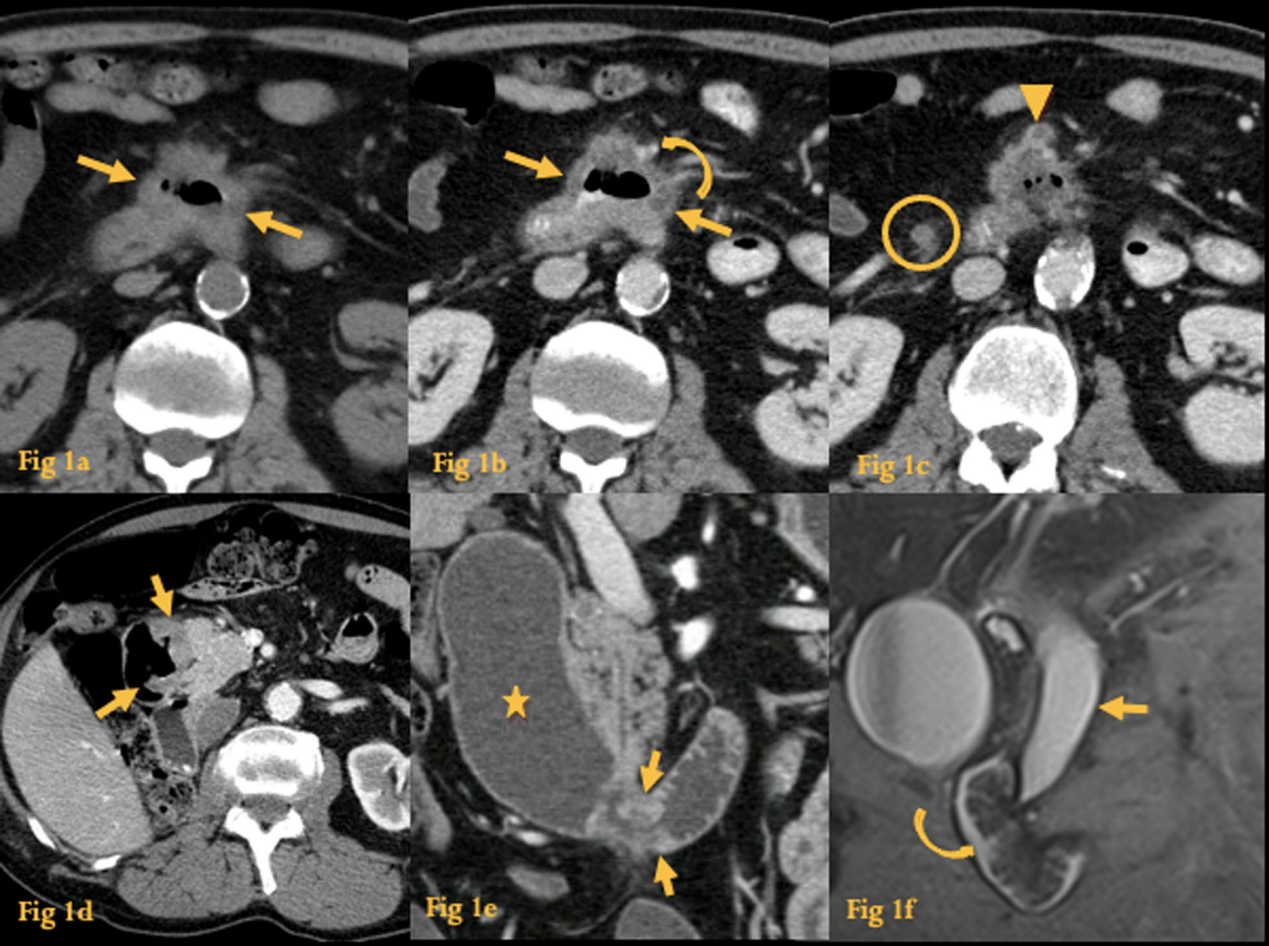
Adenocarcinoma. Abdominal CT performed before (**a**) and after (**b**, **c**) iv contrast administration shows irregular circumferential asymmetric thickening of D3, causing deformity of the lumen (arrows). There is encasement of the superior mesenteric artery (curved arrow) and tumor thrombosis of the superior mesenteric vein (arrowhead), rendering the lesion surgically unresectable. There is also periduodenal fat stranding and adenopathy (circle). Abdominal CT (**d**) of another case of duodenal carcinoma with an ulcerated mass at D2 (arrows). Abdominal CT of yet another case of duodenal carcinoma (**e**); there is circumferential duodenal thickening (arrows) which caused luminal stenosis with proximal dilatation (asterisk). MRCP of another patient (**f**) showing a polypoid duodenal lesion (curved arrow) that caused CBD dilatation (arrow), which proved to be a duodenal adenocarcinoma

CT reflects the same findings seen in the UGI series. It usually manifests as a soft-tissue attenuation thickening of the duodenal wall with enhancement (Fig. 1a–d). It most frequently arises in the periampullary area (Fig. 1d, e) and is rare in the bulb. In contrast with lymphoma, adenocarcinoma typically causes obstruction. When it involves the D2, there might be common bile duct (CBD) obstruction (Fig. 1d, e). Perilesional adenopathy is frequent at the time of diagnosis (Fig. 1a–c) and 5–40% of the patients have distant metastases or peritoneal seeding at presentation.

Differentiation between adenocarcinoma of the duodenum and other malignant tumors of the ampulla, pancreas, and common bile duct, with duodenum invasion, could be extremely difficult [2].

### Ampullary tumors

**Fig. 2:**
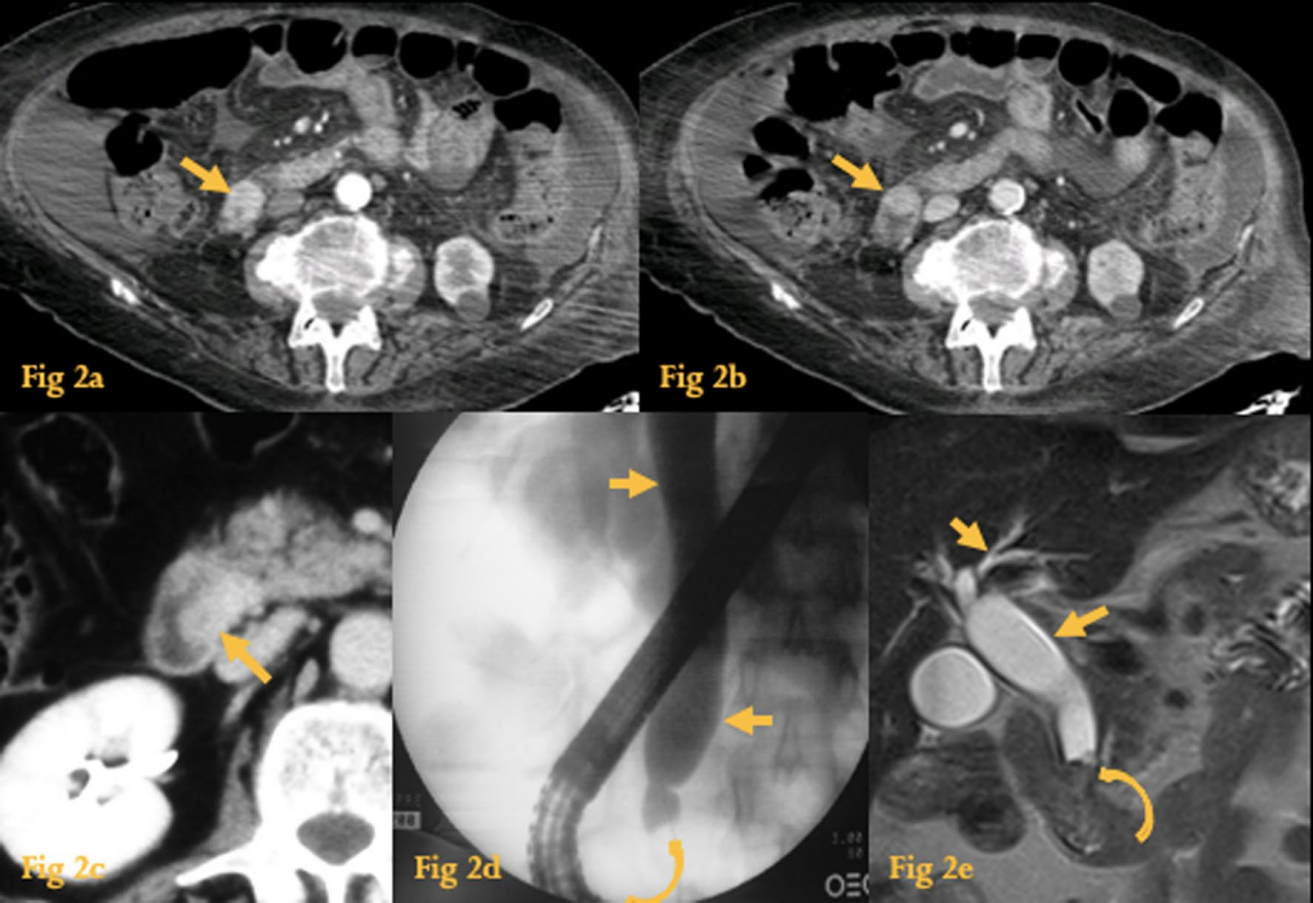
Ampullary tumors. Contrast-enhanced CT (**a**, **b**) reveals an enhancing soft-tissue mass (arrows), originating in the medial wall of the descending duodenum. This was a case of an ampullary adenocarcinoma. Contrast-enhanced CT of another patient with an ampullary carcinoma (**c**), shows a soft-tissue mass (arrow) originating in the medial wall of the descending duodenum and protruding into the water-filled duodenum. ERCP (**d**) revealed an enlarged and irregular suspicious papilla (curved arrow) and CBD dilatation (arrows). MRCP (**e**) of another patient shows dilatation of the CBD and intra-hepatic ducts (arrows) due to an abrupt stop at the distal CBD (curved arrow), which was caused by an ampullary villous adenoma

Periampullary tumors include benign and malignant neoplasms that arise within 2 cm of the ampulla of Vater, such as true ampullary tumors (Fig. 2), adenocarcinoma of the pancreatic head, cholangiocarcinoma involving the intra-pancreatic distal bile duct, and duodenal carcinoma [3]. Most periampullary tumors are adenocarcinomas.

There are two types of ampullary tumors: benign ampullary adenomas and malignant ampullary adenocarcinomas.

Because of their similar clinical and imaging characteristics (biliary and pancreatic obstruction), the differential diagnosis between true ampullary tumors and periampullary tumors is very difficult.

Endoscopic cholangiopancreatography (ERCP) and magnetic resonance cholangiopancreatography (MRCP) may be useful in determining the origin of the periampullary tumor (Fig. 2d, e).

CT may show wall thickening or a polypoid mass arising from the medial wall of D2, with associated biliary duct dilatation (more commonly), without or with pancreatic duct dilatation (double duct sign) [4].

### Afferent loop syndrome

**Fig. 3:**
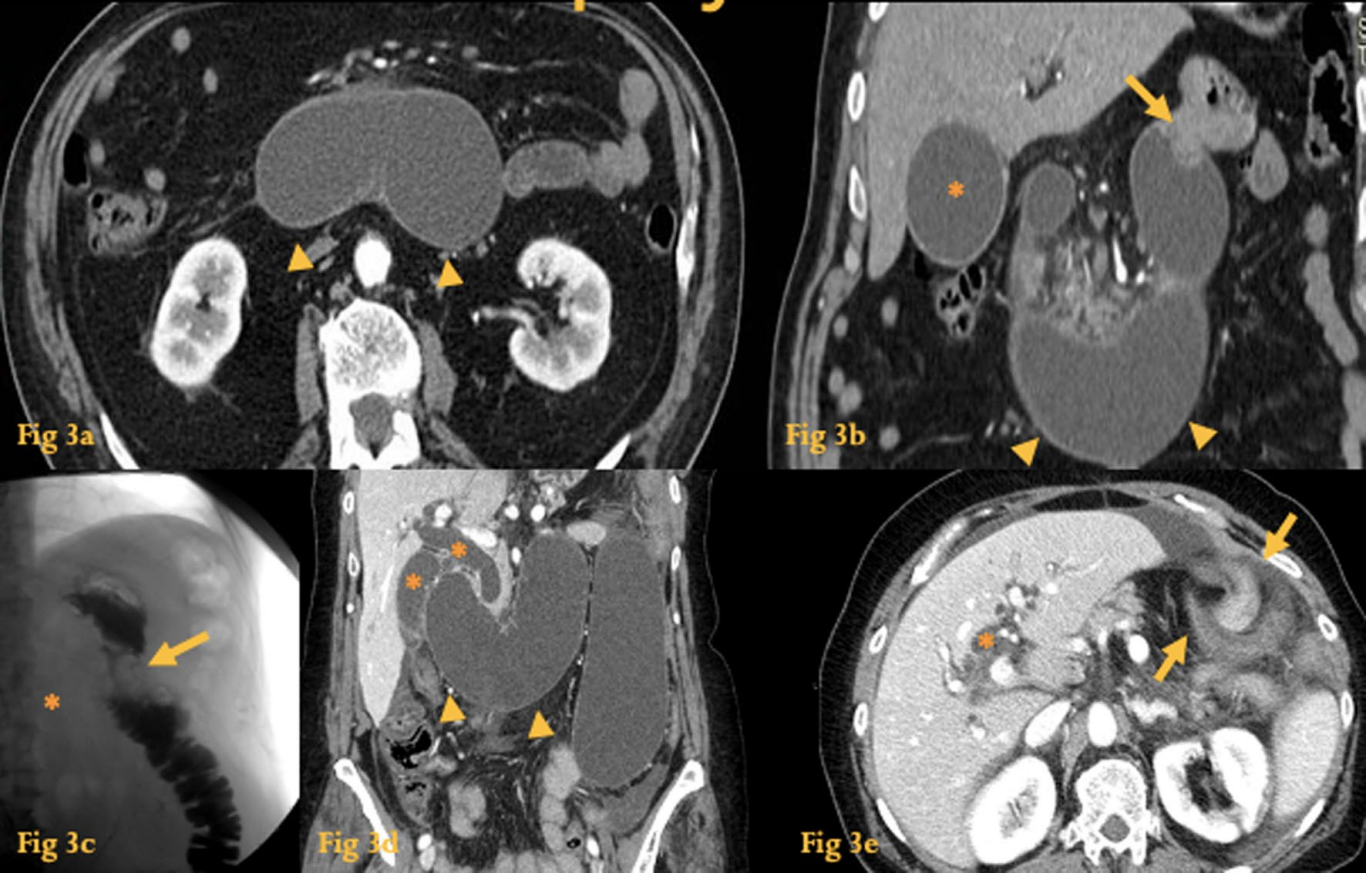
Afferent Loop Syndrome. Abdominal CT (**a**, **b**) of a patient who had received partial gastrectomy with Billroth II reconstruction—A fluid-filled tubular structure in the right upper quadrant and crossing the midline is seen, with associated gallbladder distension (asterisk). There is an enhancing lesion at the anastomosis. This was an afferent loop syndrome caused by gastric tumor recurrence. UGI series of the same patient (**c**). There is non-filling of the afferent loop (asterisk) and a filling defect at the location of the anastomosis (arrow). Abdominal CT (**d**, **e**) of another patient with afferent loop syndrome, because of an internal hernia with volvulus (note the twisted configuration of the bowel loops at the left hypochondrium (arrows)). There is fluid-filled dilatation of the afferent loop associated with gallbladder distension and intrahepatic and extrahepatic duct dilatation (asterisks)

Afferent loop syndrome (ALS) represents a mechanical obstruction of the afferent loop in 13% of the patients submitted to pancreaticoduodenectomy [5]. Most cases of ALS are due to adhesions, anastomotic strictures, recurrent tumor, and internal hernias (Fig. 3) [6].

The backpressure from the dilated afferent loop can cause biliary dilatation and acute pancreatitis.

Although UGI series can be helpful in the diagnosis by allowing visualization of non-filling of the afferent loop (Fig. 3c), non-obstructed afferent loops are not normally filled in 20% of cases, and so this examination has a low sensitivity. Preferential filling and retention of barium in a dilated afferent loop for at least 60 min is another finding consistent with ALS.

CT is the most important imaging tool in establishing the diagnosis and determining the site, degree, cause, and complications (biliary dilatation, pancreatitis, or strangulation) of ALS [7]. A fluid-filled tubular or C-shaped structure containing small air bubbles in the right upper quadrant or crossing the midline, with *valvulae conniventes* projecting into the lumen, in symptomatic patients after gastroenterostomy, makes the diagnosis.

### Biliary ileus

It occurs following subacute or chronic cholecystitis that leads to gallstone erosion into the bowel (most often through a cholecystoduodenal fistula) [8].

The site of obstruction is usually the terminal ileum because it is the narrowest portion of the small bowel.

Rigler’s triad encompasses the three typical findings of gallstone ileus on a radiograph: pneumobilia, dilated small bowel loops, and a calcified gallstone in an ectopic location.

**Fig. 4:**
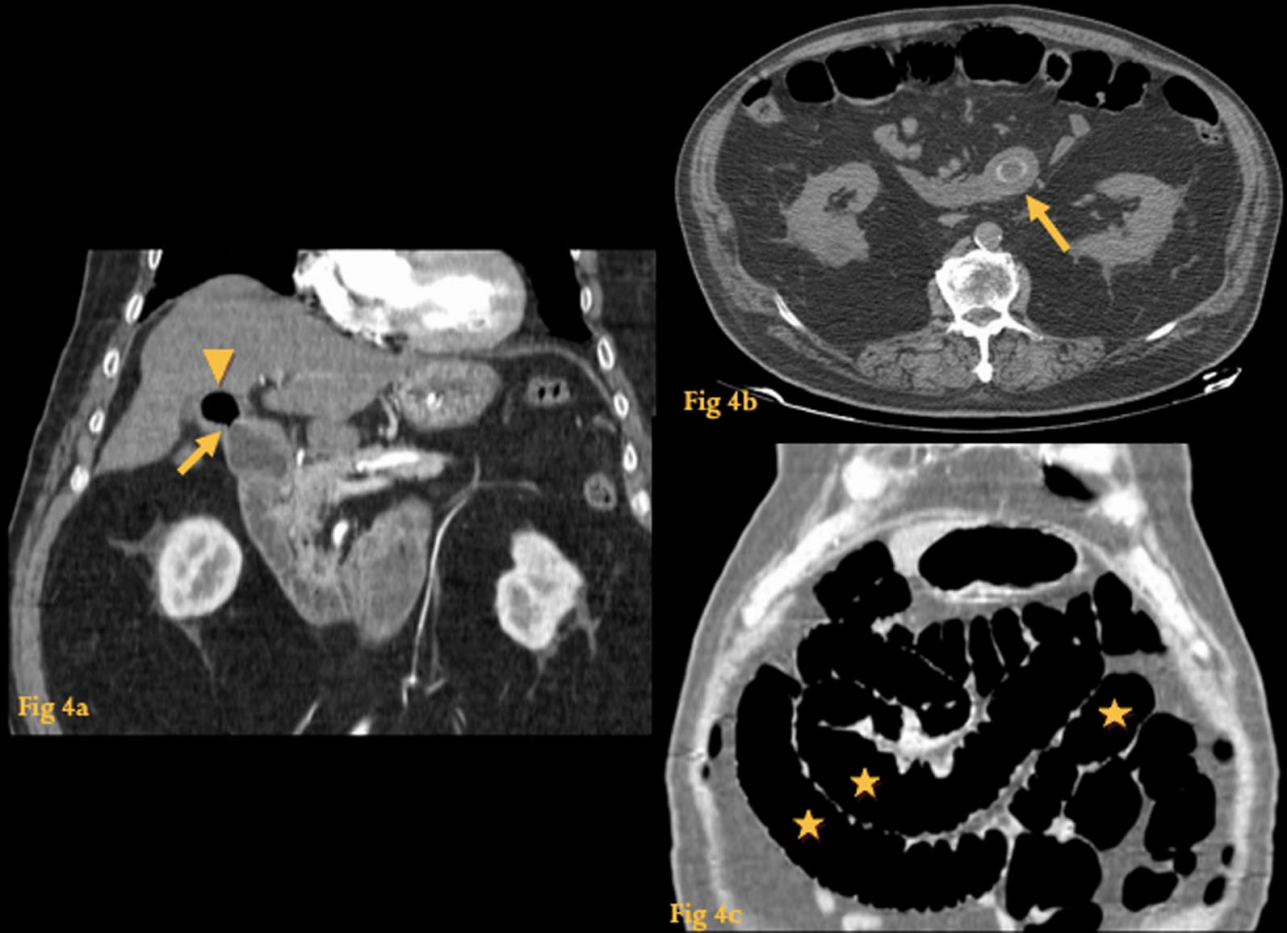
Biliary ileus. CT coronal reformation (**a**) shows air in the gallbladder (arrowhead) and communication between its lumen and D2—cholecystoduodenal fistula (arrow). Axial CT scans (**b**) show a 2 cm-wide elliptical gallstone impacted at the distal jejunum (arrow). Coronal reformation 9**c**) shows air-filled dilated small bowel loops (asterisk)

CT is the modality of choice because it allows a correct diagnosis of gallstone ileus with higher accuracy and detects the exact location of the ectopic stone and the biliary–enteric fistula [9] (Fig. 4).

### Bouveret syndrome

Bouveret syndrome is a subtype of gallstone ileus which refers to gastric outlet obstruction due to the impaction of a gallstone in the pylorus or proximal duodenum [10].

The diagnosis was usually made with endoscopy but is increasingly being found on CT in the setting of abdominal pain in emergency departments.

On the UGIS, there is an endoluminal filling defect in the duodenum that produces partial or complete gastric outlet obstruction.

**Fig. 5:**
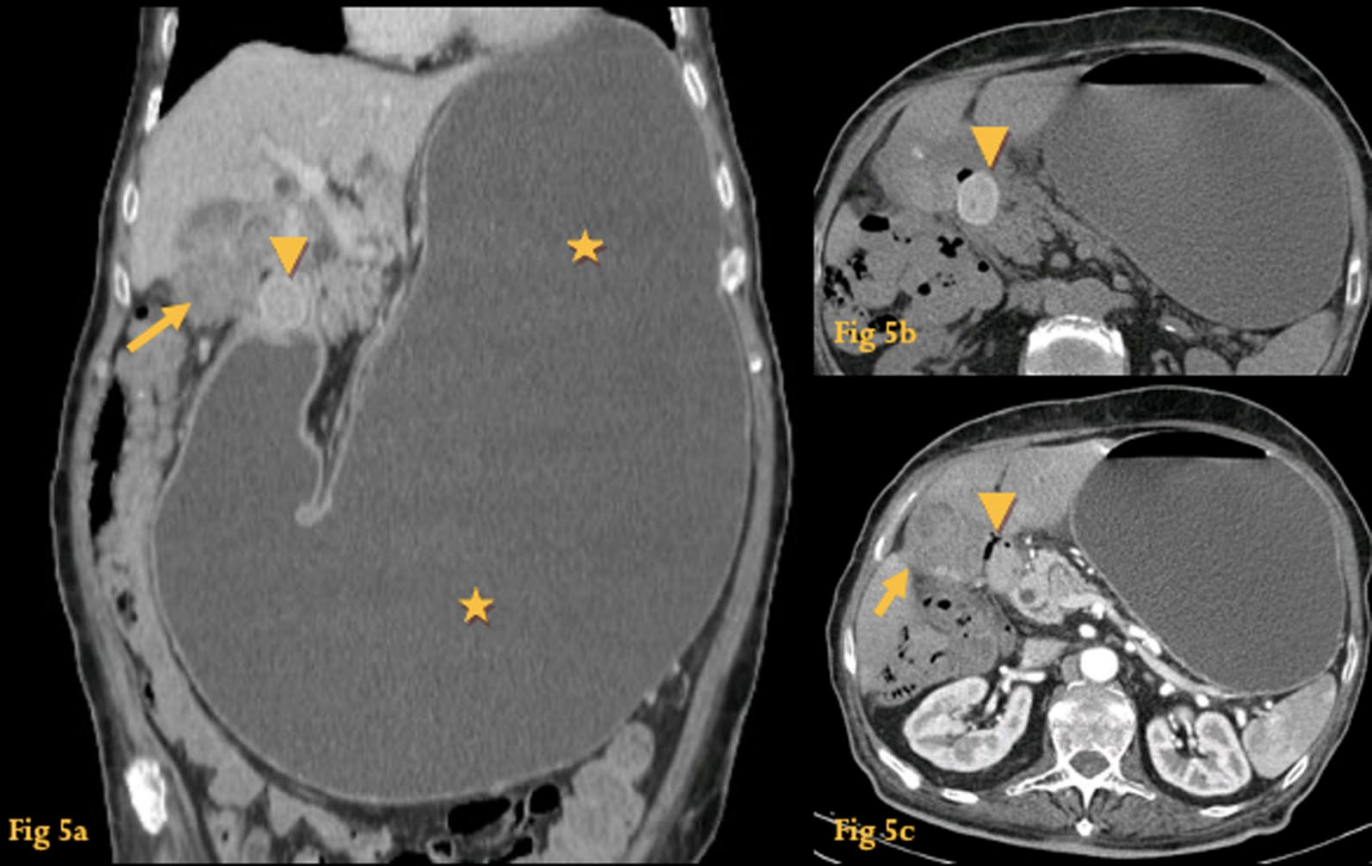
Bouveret syndrome. CT coronal reformation (**a**) shows marked gastric dilatation (asterisks) caused by a round lesion at the duodenal bulb (arrowhead); there is also densification and indefinition of the contour of the gallbladder (arrow). Unenhanced CT (**b**) shows the lesion is spontaneously hyperdense-endoluminal gallstone at the duodenal bulb (arrowhead). A small fistulous tract filled with air (**c**) (arrowhead) is seen between the duodenal bulb and the expected location of the gallbladder, which had ruptured and formed a phlegmon (arrow)

CT shows to a better extent the gallstone, the cholecystoduodenal fistula, and the gastric dilatation that make up this syndrome (Fig. 5).

### Cystic dystrophy of the duodenal wall

**Fig. 6:**
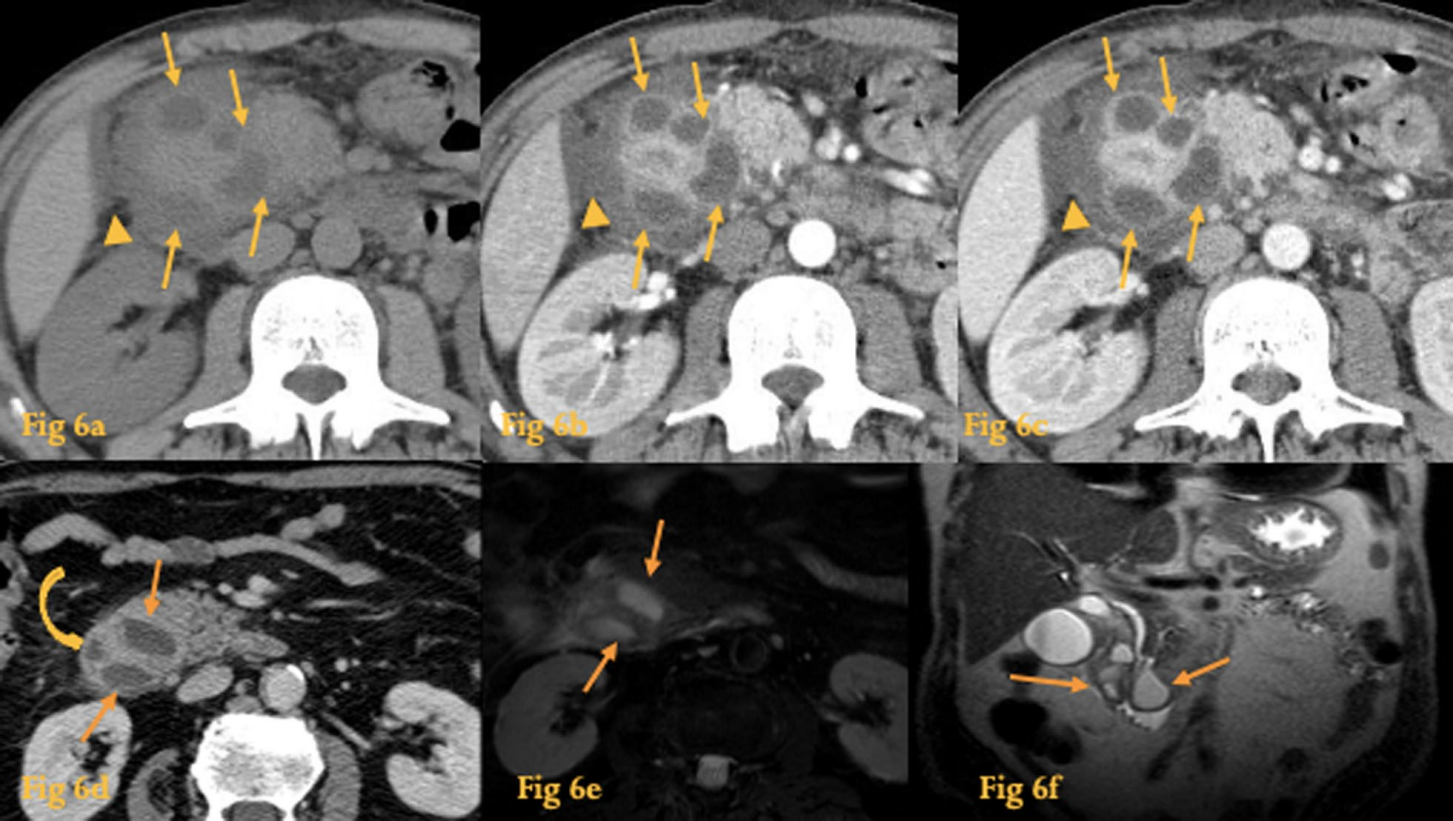
Cystic dystrophy of the duodenal wall. Abdominal CT of a patient with abdominal pain (**a**–**c**)—there is parietal thickening and several cystic lesions surrounding the duodenum (arrows), best evaluated after contrast administration (**b**, **c**). These cysts have a preferential localization in the mesenteric side. Densification of the periduodenal fat and a small amount of fluid surrounding the duodenum (arrowheads) is also seen. Abdominal CT of another patient (**d**)—duodenal intramural cyst-like lesions with fluid density (arrows). The cysts narrow and shift the duodenal lumen to the right. In abdominal MR (**f**, **e**), cystic lesions (arrows) present high signal intensity on T2w images

Cystic dystrophy of the duodenal wall is related to groove pancreatitis [11] and is now considered to be part of the spectrum of paraduodenal pancreatitis (see letter P for paraduodenal pancreatitis). It is characterized by the presence of heterotopic pancreatic tissue within the duodenal wall [12]. The pancreatic duct's obstruction of this ectopic pancreas results in an inflammatory process and cystic lesions in the duodenal wall (Fig. 6).

Two patterns are described according to the lesion size: the more common cystic pattern, in which the cysts are > 1 cm (may mimic an intraluminal diverticulum) and the uncommon solid pattern, in which the lesions are < 1 cm (may simulate a duodenal neoplasm).

The cysts are usually located within the thickened submucosa or muscular layers, of the medial wall of the descending duodenum.

CT shows multiple small and elongated cysts within the duodenal wall, mural thickening of the descending duodenum, with or without luminal stenosis [12].

MRCP can aid in the diagnosis by displaying cystic changes along the duodenal wall and in the pancreatico-duodenal groove.

### Choledochocele

Choledochal cysts are rare congenital dilatations of the common bile duct. The diagnosis of a choledochal cyst is made based on the disproportional dilatation of the extrahepatic bile ducts after excluding mechanical factors (stones, inflammation, or tumors) as the cause of the dilatation.

**Fig. 7:**
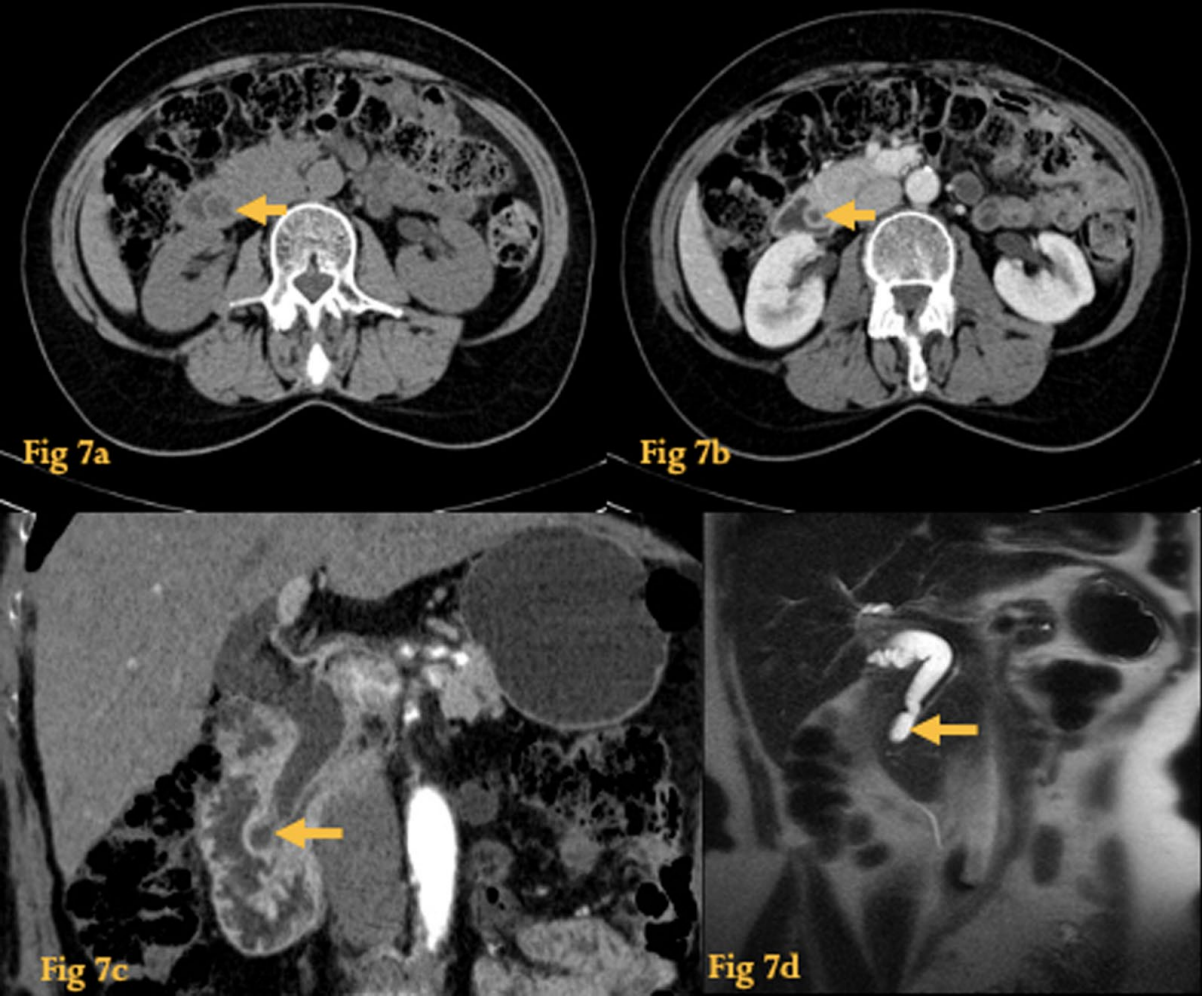
Choledococele. Unenhanced (**a**) and enhanced CT (**b**) shows a fluid-filled sac within the medial wall of the descending duodenum. CT coronal reformation (**c**) shows protrusion of a dilated distal segment of the common bile duct into the duodenum. Abdominal MR (**d**), coronal HASTE heavily T2-weighted sequence, shows distal CBD dilatation which protrudes into the duodenum

Choledochocele is a cystic/diverticular herniation of the distal common bile duct into the duodenum (Fig. 7) [13]. It represents a type III choledochal cyst under the Todani classification and they comprise 2% of all choledochal cysts [14]. Some authors consider this entity as a variant of duodenal duplication.

CT shows a fluid-filled sac that protrudes into the duodenal lumen but does not opacify when oral contrast is given. Complications of choledochal cysts such as cholecystitis, cholangitis, biliary stricture, choledocholithiasis, pancreatitis, and malignant transformation into cholangiocarcinoma are promptly diagnosed with CT. MRCP may help when CT is doubtful (Fig. 7d).

### Duplication cyst

**Fig. 8:**
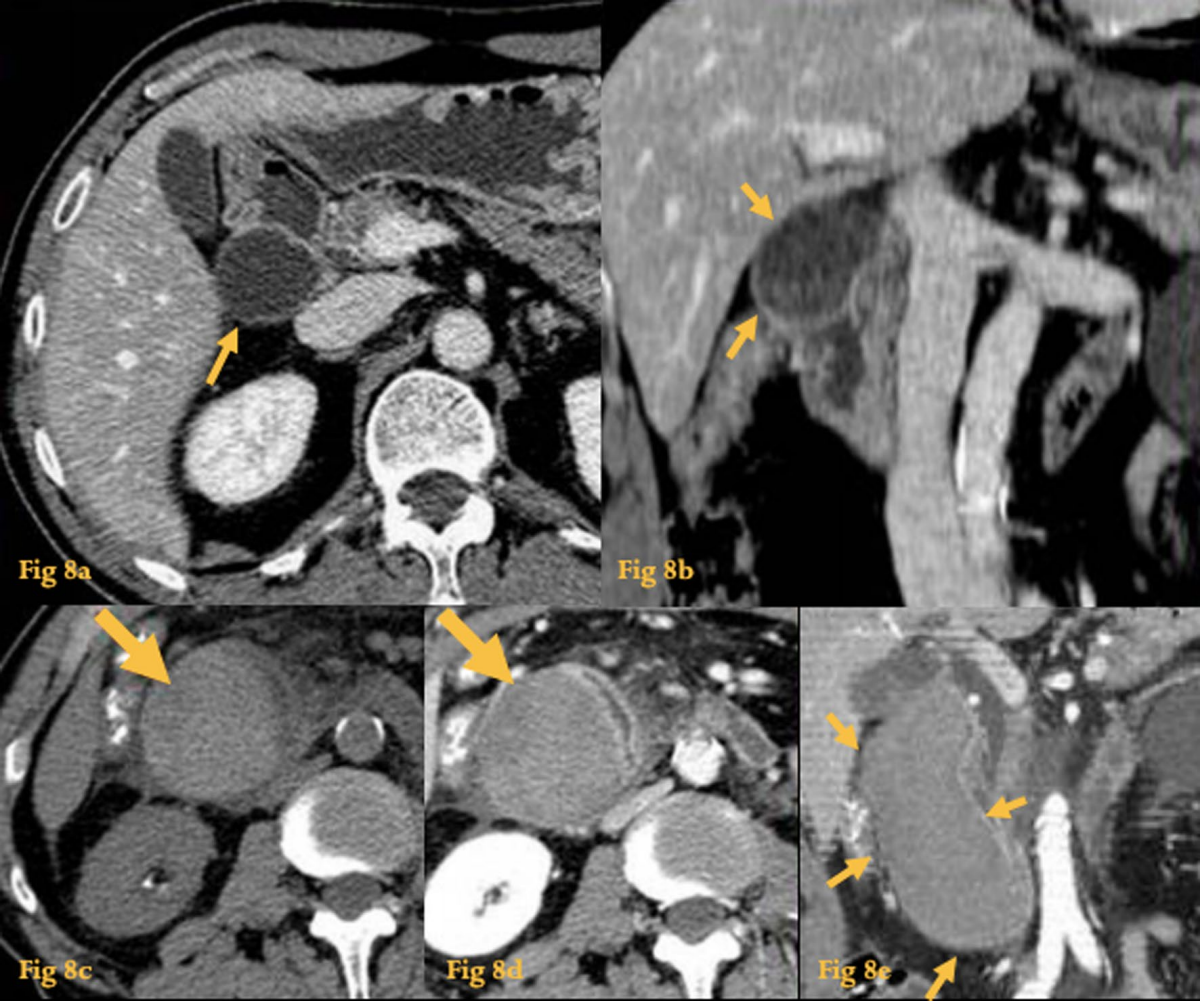
Duplication cyst. Abdominal CT (**a**, **b**) fortuitus finding of a peri-duodenal cystic lesion (arrows), with mural enhancement, which caused extrinsic compression on the descending duodenum. Abdominal CT of the same patient some months after (**c**–**e**), when he started complaining of abdominal pain, shows increasement in size and spontaneously hyperdense content on unenhanced images (arrow on **c**). Histopathological study of the resected specimen revealed a duodenal duplication cyst complicated with hemorrhage

GI duplication cysts most often originate from the small bowel. Duodenal duplication cysts most commonly arise in the medial wall of the D2 and D3. Most are incidental findings at CT (Fig. 8a, b). Some of them may contain gastric or pancreatic tissue, and so may ulcerate or hemorrhage [15].

Because most duplication cysts do not communicate with the bowel lumen, on CT they typically appear as a well-circumscribed cystic mass with fluid attenuation. Intracystic hemorrhage may occur (Fig. 8c–e). The presence of mural nodules should raise the concern of a carcinoma arising from the duplication cyst, which is an extremely rare situation [4].

Endoscopic ultrasonography (EUS) is the diagnostic tool of choice to investigate duplication cysts since it can distinguish between solid and cystic lesions and establish cyst location relative to surrounding tissues [16].

### Diverticula

Duodenal extramural diverticula are incidental findings in most of the patients.

They may result from mucosal prolapse or the prolapse of the entire duodenal wall [4] and are most frequently found on the inner aspect of D2, adjacent to the ampulla of Vater.

Duodenal diverticula may present with serious complications like perforation, duodenal fistulas, intra-abdominal abscesses, and sepsis.

**Fig. 9:**
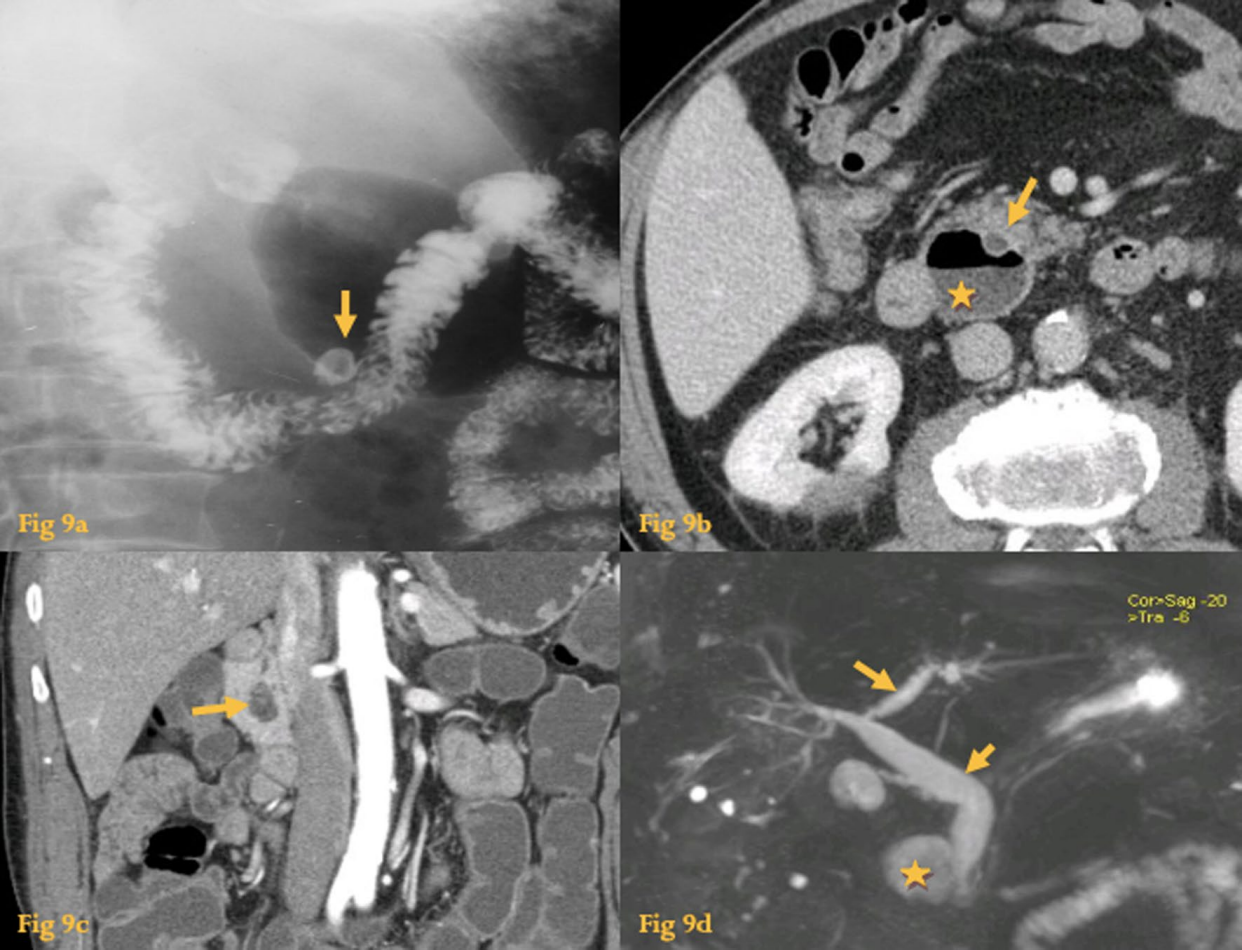
Diverticula. Upper gastrointestinal series with barium contrast (**a**) shows an outpouching of the upper wall of the third part of the duodenum (arrow). Abdominal CT (**b**) shows an air and fluid-filled diverticulum on the inner aspect of the descending duodenum (asterisk), adjacent to the CBD (arrow). In another patient (**c**), an heterogeneous fluid and air-filled diverticulum on the inner aspect of the descending duodenum (arrow) mimicked an abscess. MRCP (**d**) in a patient that had bile duct dilatation (arrows) with associated cholangitis due to a periampullary diverticulum (asterisk)

UGIS show barium-filled pouch-like projections beyond the normal lumen and can be differentiated from ulcers by visualization of mucosal folds entering the neck of the diverticulum and change in appearance with peristalsis (Fig. 9a).

CT shows as a fluid and/or gas-filled collection in a typical location, that communicate with the duodenal lumen (Fig. 9b). A duodenal diverticulum that is entirely filled with fluid may mimic a cystic neoplasm/pseudocyst arising from the pancreas. If it also has gas, differential diagnosis with an abscess is in order (Fig. 9c).

Periampullary diverticula rarely can cause bile duct obstruction (Fig. 9d) with jaundice and cholangitis and / or pancreatitis [17] – Lemmel syndrome.

### Ectopic pancreas

Ectopic pancreas is defined as abnormally located pancreatic tissue with its own ductal system, with no vascular, neural, or anatomic contact with the normal gland [18].

**Fig. 10:**
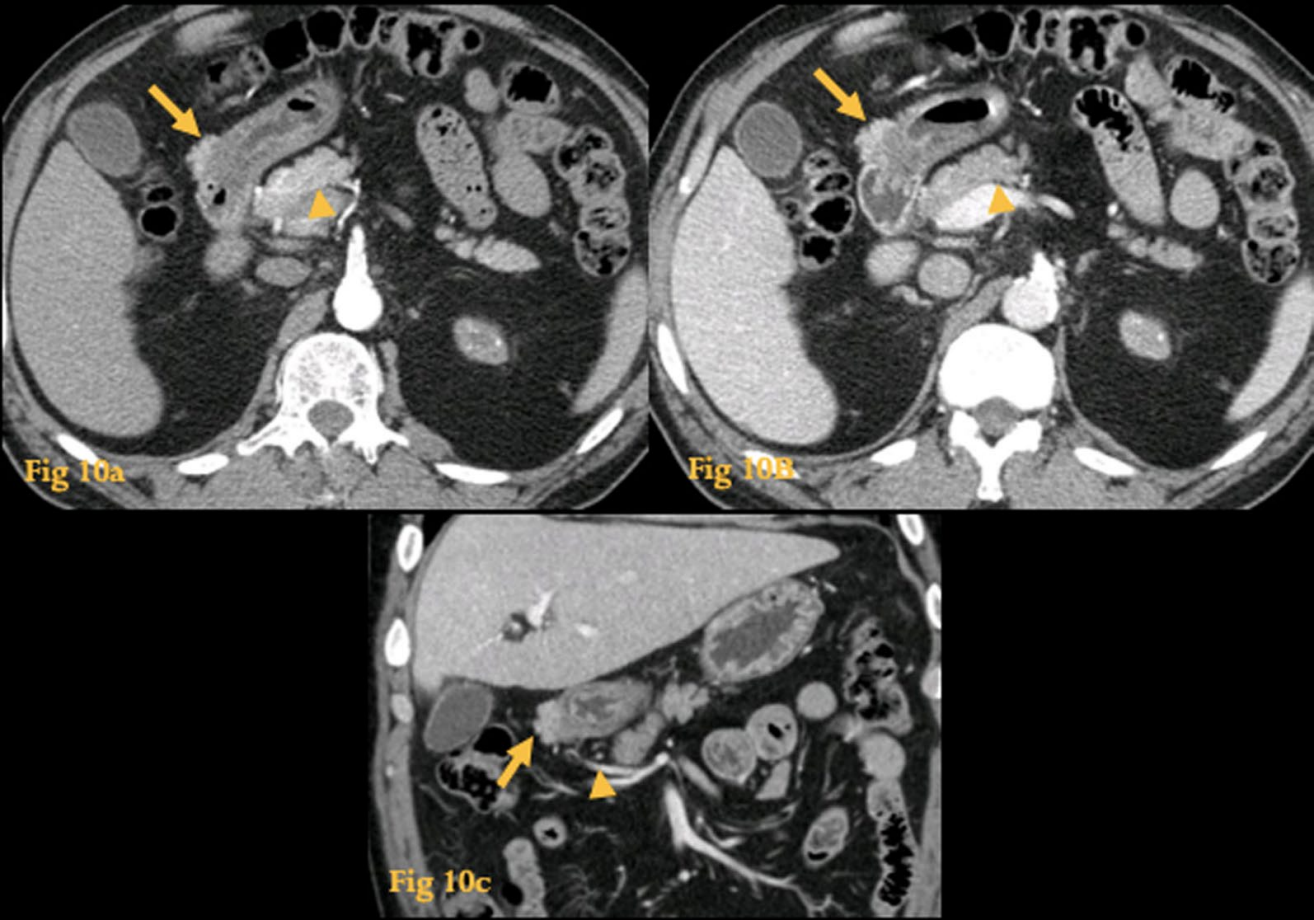
Ectopic pancreas. Abdominal CT of a patient who underwent the exam for an unrelated reason. A soft-tissue nodule, with a flat-ovoid shape and microlobulations, is seen in the serosa of the first portion of the duodenum. It has an enhancement pattern similar to that of the normal pancreatic tissue (arrowheads on **a**, **b**) in the late arterial (**a**) and portal venous phases (**b**). The lesion (arrow) is supplied by a branch of the gastroduodenal artery (arrowhead on **c**). This was heterotopic pancreatic tissue in the duodenal bulb serosa

It is the most common heterotopia within the gastrointestinal system and is usually an incidental finding in CT scans or autopsy (Fig. 10). Nevertheless, the same pathology that affects the normal gland may also be seen at this ectopic site.

The most common locations are the duodenum (28%), stomach (26%), and jejunum (16%). It most frequently occurs in the submucosa (75%), but can also be present within the muscularis propria or the serosa.

On UGIS, a characteristic appearance is a central small collection of barium inside an umbilicated nodule in the duodenal submucosa.

On CT, presents as a mass in the duodenal wall with an enhancement pattern similar to that of pancreatic tissue. GIST and leiomyoma have to be differentiated from this condition because they all share a submucosal origin. A flat shape, lobulated contour, and endoluminal growth are most indicative of ectopic pancreas.

MRCP may depict the presence of ducts within the tissue, a finding that is pathognomonic for ectopic pancreas [19].

### Extension from adjacent tumor

Secondary involvement of the duodenum with malignancy can occur by local extension from adjacent sites or metastases from distant organs [4].

**Fig. 11:**
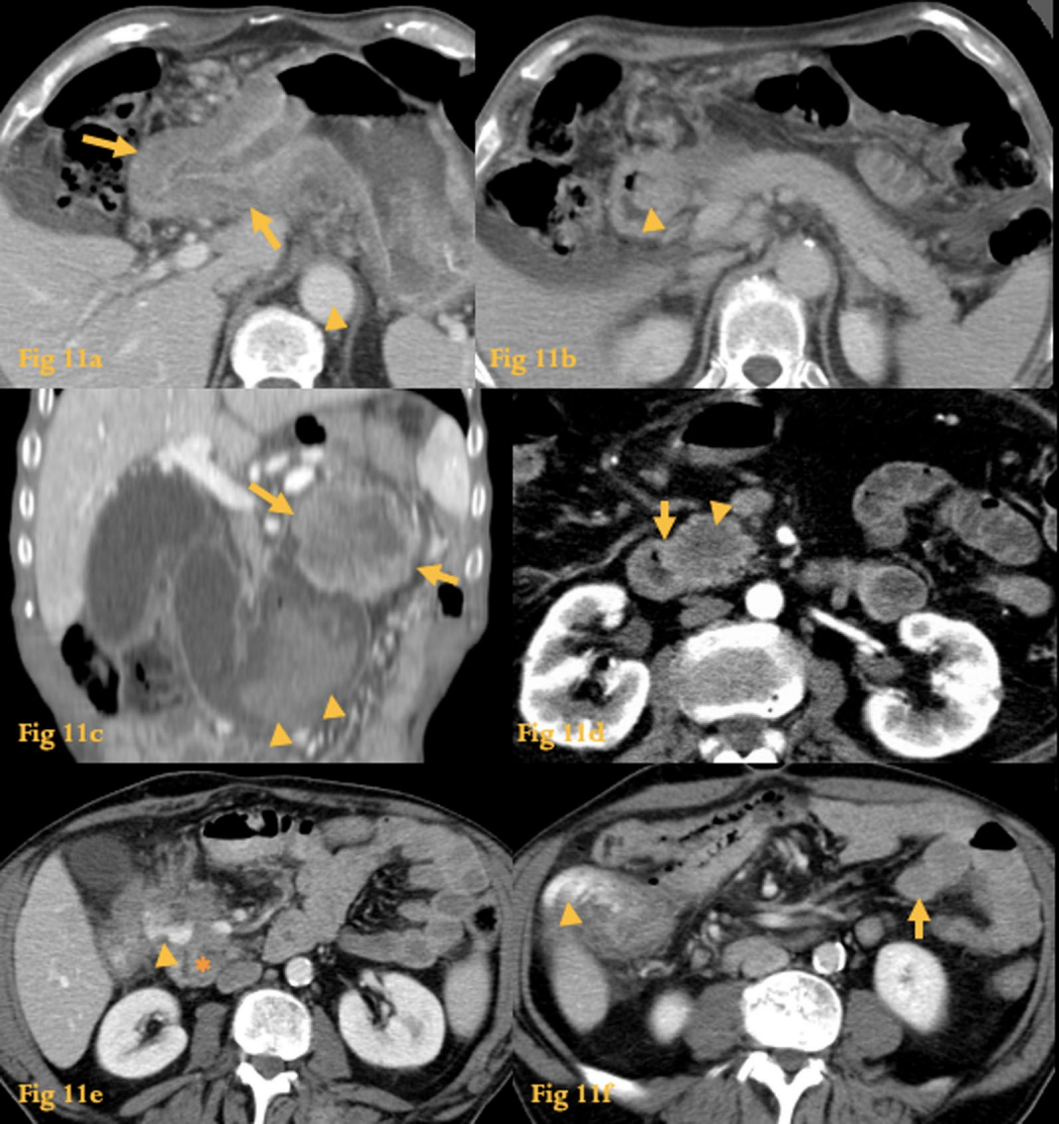
Extension from adjacent tumor. Abdominal CT (**a**, **b**) shows circumferential wall thickening of the gastric antrum (arrows) extending through the pylorus into D1 and D2, where a polypoid-like lesion (arrowhead) narrows the lumen. CT of another patient (**c**) shows evidence of gastric adenocarcinoma (arrows) with duodenal invasion and hemorrhage (arrowheads) inside the duodenal lumen. Abdominal CT of other patient (**d**) shows a hypodense soft-tissue density mass (arrowhead) which was a pancreatic adenocarcinoma with invasion of the medial wall of D2 (arrow). Abdominal CT of a patient with a neoplasm of the hepatic flexure of the colon (**e**, **f**). An undefined mass apparently involves the duodenum (asterisk) and the colon. A small amount of positive oral contrast agent (arrowhead) is present within the mass. An image obtained caudally (**f**) shows oral contrast agent in the colon (arrowhead) without evidence of contrast in the jejunum (arrow), a feature related to a colo-duodenal fistula

Gastric carcinomas may extend through the pylorus into the duodenal bulb or invade D4 (Fig. 11). Gastric adenocarcinoma recurrence at the anastomosis may cause afferent loop syndrome (*see letter A*).

Pancreatic adenocarcinomas can be locally aggressive, and extension to the duodenum is sometimes seen at CT [20].

Colon carcinoma may cause local mass effect or duodenal invasion, occasionally with the formation of a colo-duodenal fistula.

### Fistula (aorto-duodenal)

An aorto-duodenal fistula should always be suspected in patients who have undergone aortic graft surgery and present with gastrointestinal bleeding (specially hematemesis).

Direct communication between the duodenum and the aorta is one of the most feared clinical situations of vascular surgery, with a very high fatal outcome (near 100% if not promptly operated). The most common locations are the D3 and D4 because these are the sites closer to the aorta.

These fistulas can be primary (in a diseased segment of the aorta, usually a mycotic aneurysm) or secondary (after aortic surgery and/or the placement of a synthetic aortic graft). Since there has been an increase in this kind of surgery, the secondary type is now more common than the primary one.

**Fig. 12:**
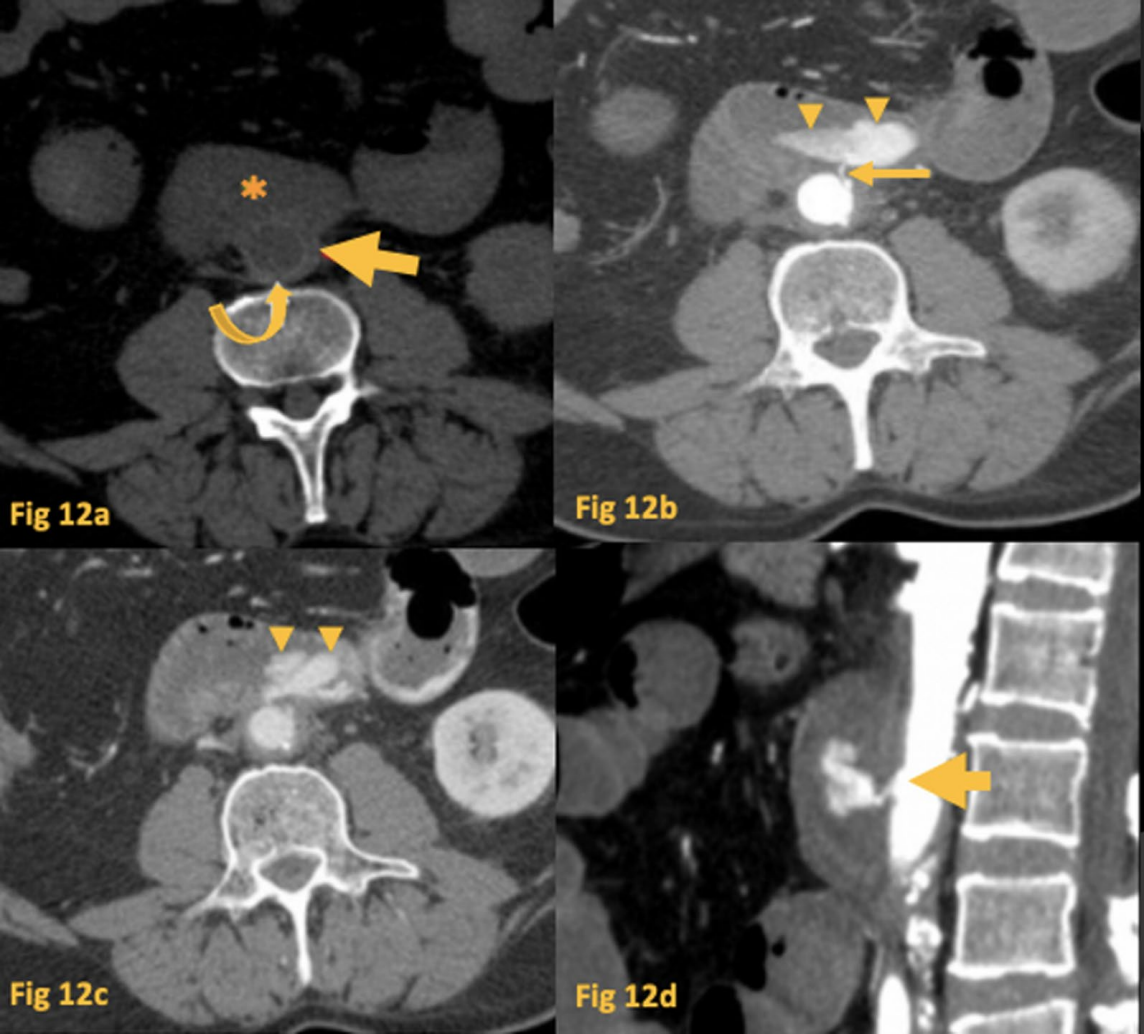
Fistula (Aorto-duodenal). Unenhanced CT (**a**) of patient with hematemesis shows an aorto-bi-femoral stent (curved arrow) inside the infra-renal aorta, with exclusion of atherosclerotic plaque (arrow), in close proximity to the third part of the duodenum (asterisk). Arterial phase CT (MIP) (**b**) shows an aortoduodenal fistula (arrow), with blood leaking into D3 (arrowheads) in the topography of the proximal end of the aortic stent. Portal phase CT (**c**) shows increased pooling of blood in the duodenum and jejunum (arrowheads). Sagittal reformat in arterial phase CT (**d**) better shows the fistulous tract of the blood into D3 (arrow)

CT is the mainstay of diagnosis (Fig. 12). The direct signs of this situation are the presence of vascular contrast inside the GI tract, ectopic gas adjacent, and within the aorta and visualization of the fistulous tract itself. Indirect signs are duodenum wall thickening, disruption of the aortic fat cover, retroperitoneal hematoma, and increased perigraft soft tissue [21].

### GIST

**Fig. 13:**
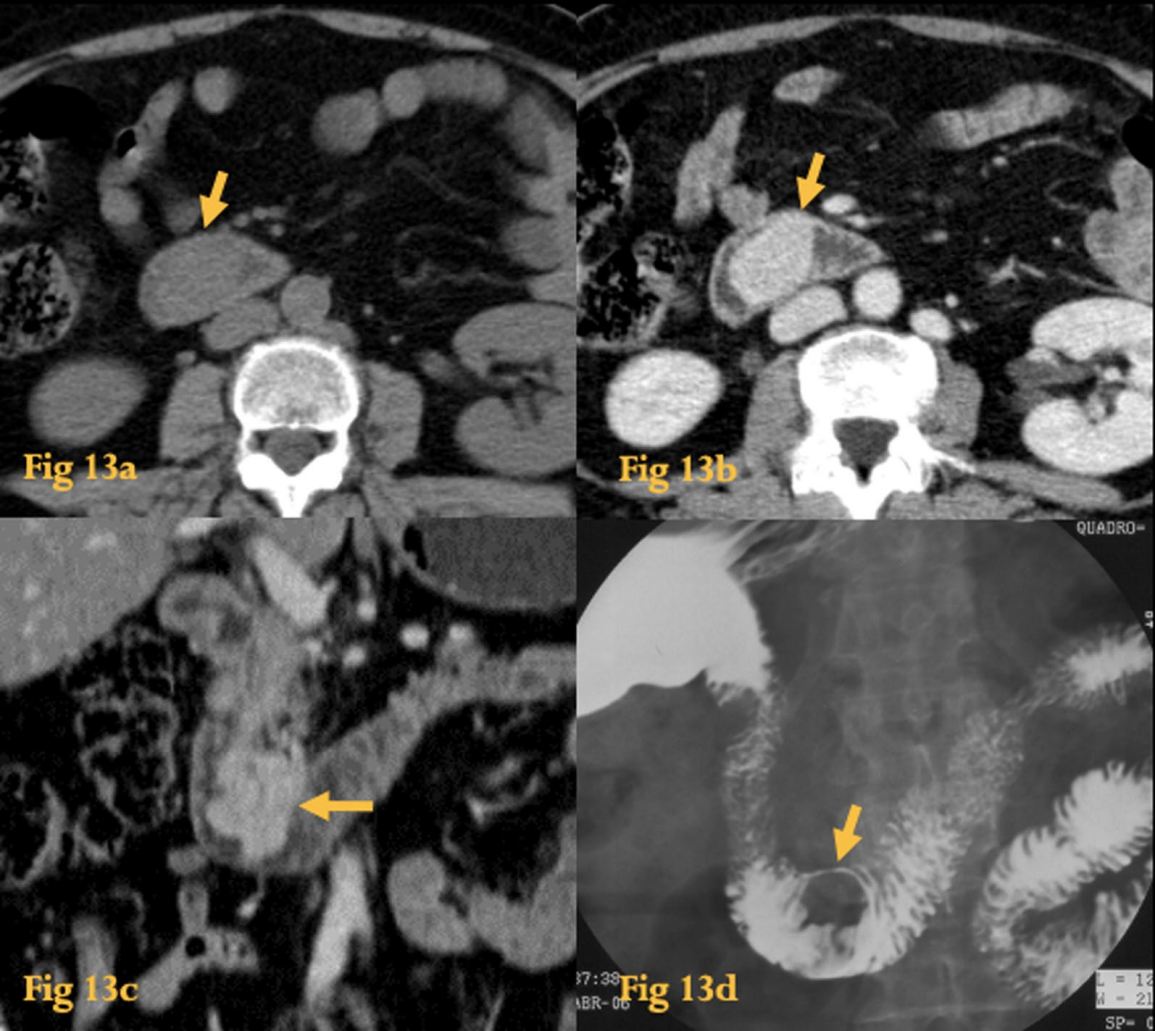
GIST. Unenhanced abdominal CT (**a**) shows an endoluminal homogeneous soft-tissue lesion on the transition D2–D3. Abdominal CT with iv contrast (**b**, **c**) shows homogeneous enhancement of the lesion (arrow). Barium study (**d**) shows a well-circumscribed round intramural mass (arrow) in the transition D2–D3. Histopathological study of the resected specimen revealed a benign GIST

GIST stands for gastrointestinal stromal tumor, which is the most common benign tumor of the small bowel (Fig. 13).

It arises from the interstitial cells of Cajal and expresses a tyrosine kinase growth factor—c-KIT (CD117) [22].

Most of GISTs are benign (70–80%). Benign behavior is particularly common in gastric tumors. Tumors of the small-bowel, including the duodenum, are prone to a more aggressive behavior [23]. They most commonly arise in the bowel wall, usually from or between the muscularis propria and muscularis mucosa.

On UGIS, the mass is typically exophytic but may also be intramural or intraluminal (Fig. 13d).

On CT, they are seen as round lesions with patchy/heterogeneous enhancement and central areas of low attenuation (necrosis or cyst formation). Smaller tumors rarely necrose and thus have homogeneous soft-tissue attenuation. Hemorrhage may occur, while calcification is uncommon. It is difficult to differentiate a benign from a malignant GIST from imaging and histologic features [23].

In general, tumors greater than 5 cm have a greater chance of being malignant.

**Fig. 14:**
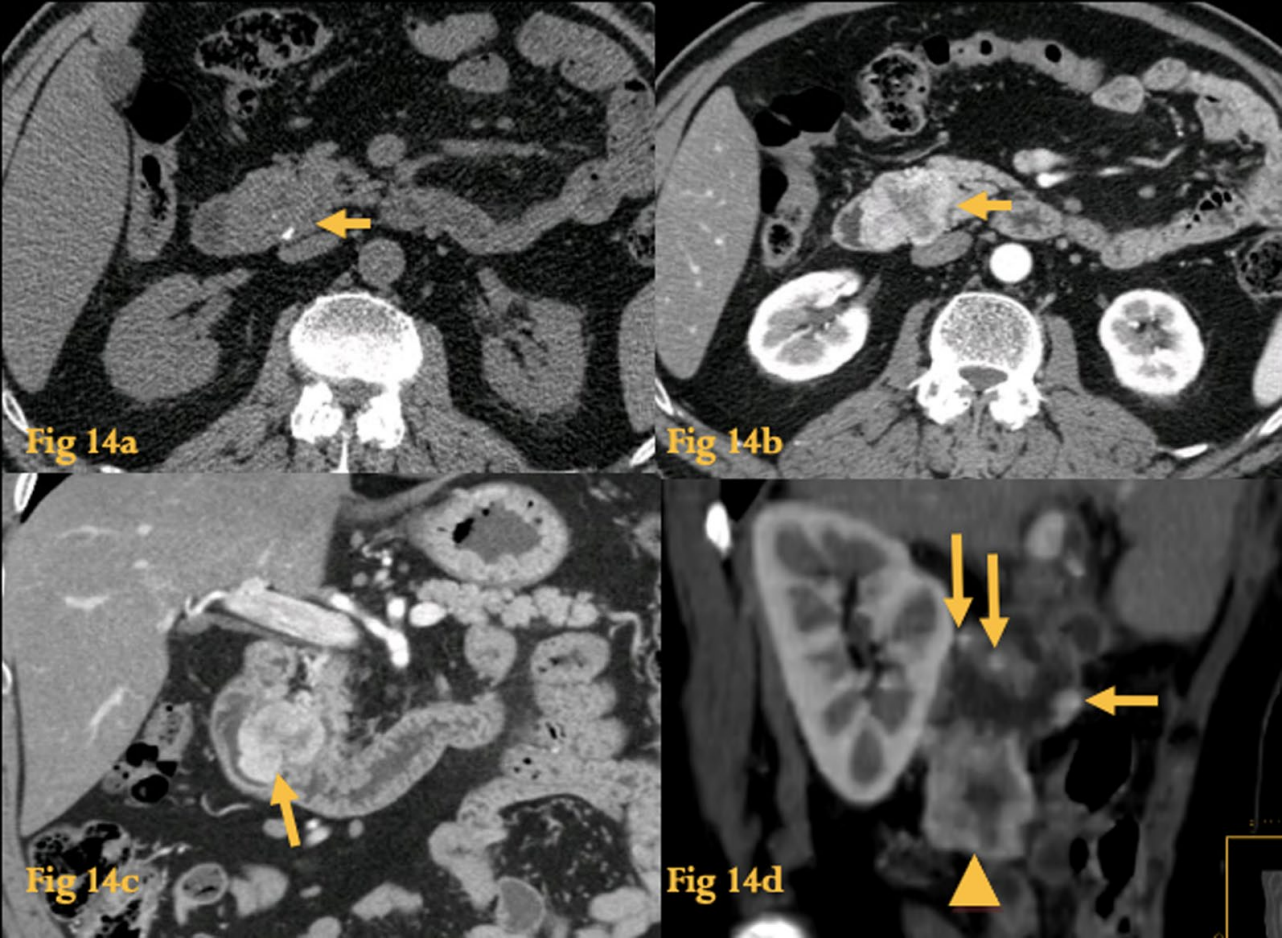
GIST. Unenhanced abdominal CT (**a**) shows a heterogeneous soft-tissue lesion with punctiform calcifications on the horizontal portion of the duodenum (arrow). Abdominal CT after iv contrast (**b**) shows peripheral enhancement and central necrosis (arrows). Coronal reformation of the same patient (**c**) better depicts the origin of the lesion (arrow) from the duodenal wall and the endophytic component. Histopathological study revealed a duodenal malignant GIST with pancreatic invasion, central necrosis, and small calcifications. Abdominal CT (**d**) of another large malignant GIST with exophytic growth pattern (arrowhead), which also shows several other small enhancing parietal metastases, in D2 (arrows)

Tumor necrosis is seen in both benign and malignant GISTs (Fig. 14b).

The most specific sign of malignancy is the presence of synchronous metastases, with the liver and peritoneum most commonly affected (Fig. 14d). Since GISTs don’t usually spread via the lymphatics, there is generally no associated lymphadenopathy.

### Hamartoma of Brunner’s glands

Brunner gland hamartoma is a very rare condition [24].

It is a proliferation of the Brunner’s glands, which are located mainly in the duodenal bulb and proximal duodenum, at the submucosa layer. Its pathogenesis and pathology remain unclear; it may evolve to adenoma and adenocarcinoma.

They are variable in size, typically 1–3 cm. Intussusception has been described in a few cases.

On UGIS, there is a nonspecific sessile or pedunculated polypoid-filling defect in the duodenum, pedunculated in the majority of cases.

**Fig. 15:**
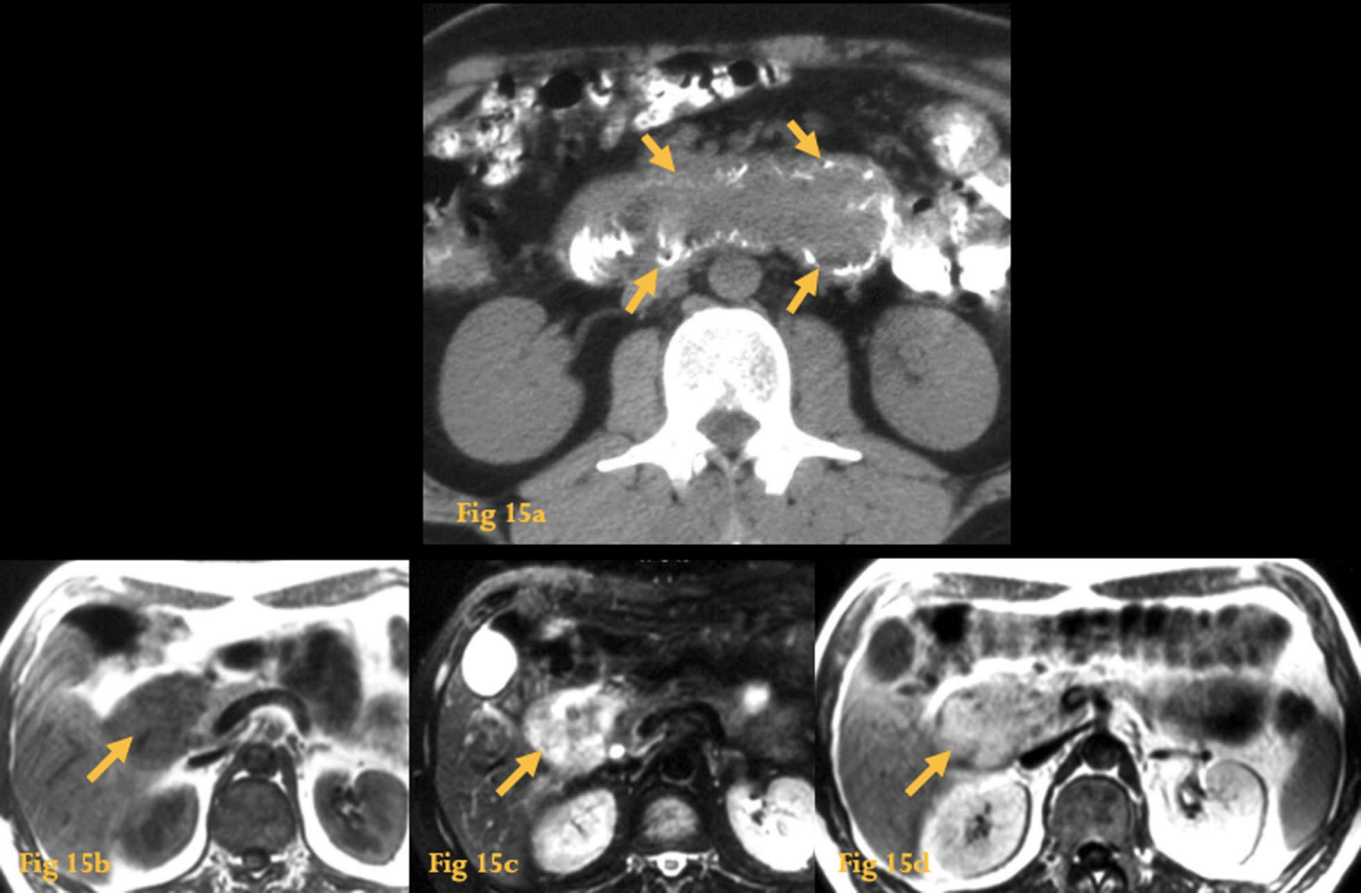
Hamartoma of Brunner’s glands. Unenhanced CT (**a**) shows a voluminous polypoid lesion originating in the duodenal bulb and reaching D4, with soft-tissue density and a lobulated contour (arrows). MRI of the same patient shows that the lesion (arrows) is heterogeneously hypointense on T1w images (**b**), hyperintense on FS T2w images (**c**), with enhancement after iv administration of gadolinium (**d**)

On CT imaging, a nonspecific polypoid lesion with submucosal origin in the duodenum is present (Fig. 15).

These lesions must be differentiated from adenomas, leiomyomas, neurogenic tumors, aberrant pancreatic tissue, prolapsed pyloric mucosa, and cystic dystrophy of the duodenal wall.

### Intussusception

Intussusception is an important cause of acute abdomen in children, where no lead point can usually be identified.

In adults, however, a specific lead point is identified in more than 90% of cases, and a neoplasm is the culprit in 65%.

**Fig. 16:**
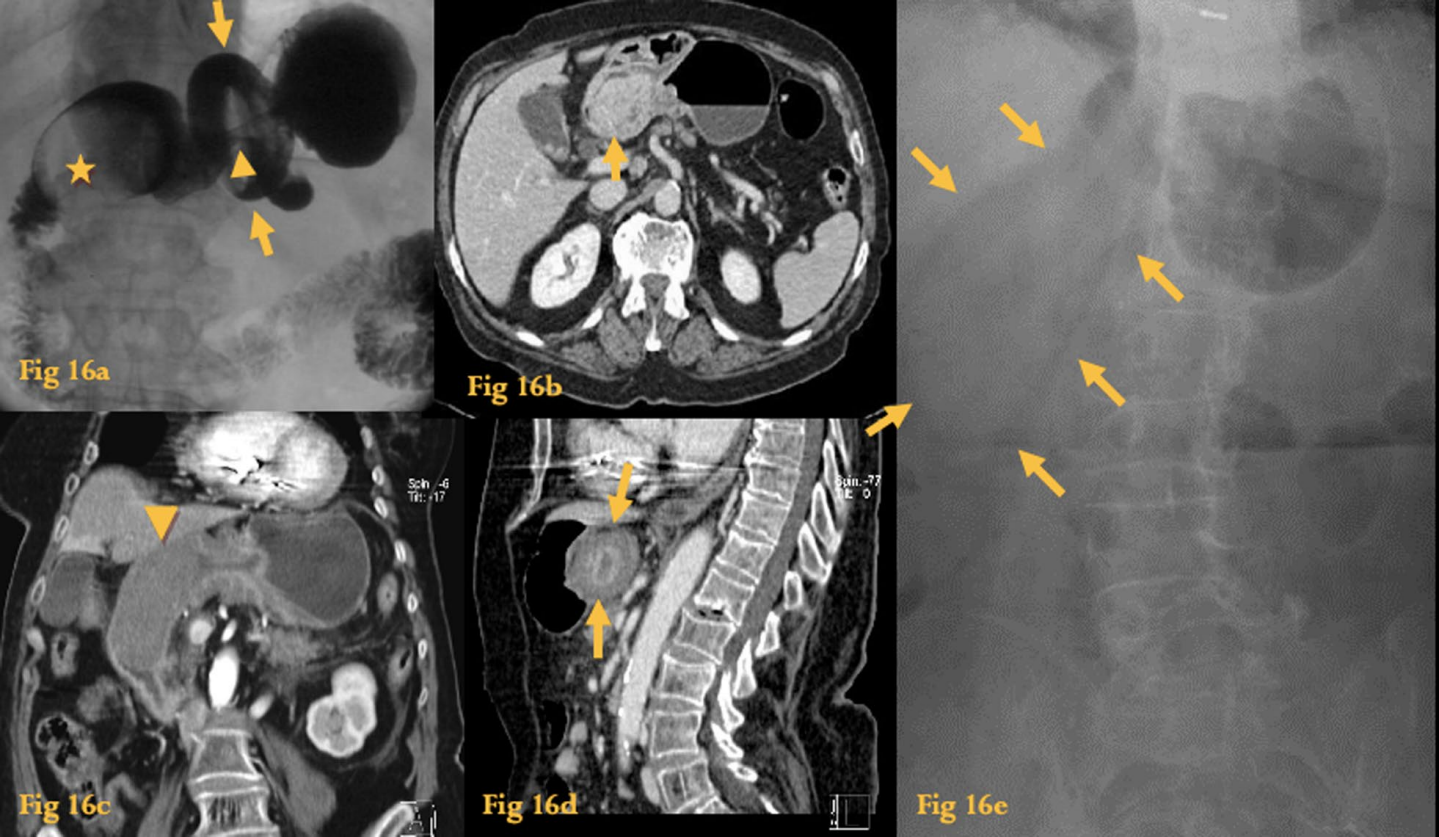
Intussusception. UGIS (**a**) rounded filling defect in the lumen of the duodenal bulb (asterisk), with regular outlines. The telescoping of mucosal folds (arrowhead) and the prepyloric collar-shaped outpouching (arrows) indicates this is an intussusception of a tumor from the gastric antrum. Abdominal CT (**b**) of the same patient with a prolapsing polypoid soft-tissue density mass with slightly heterogeneous enhancement (arrow). This was a gastric GIST. Abdominal CT of another gastric GIST (**c**, **d**) prolapsing to the duodenum (arrowhead). The coronal reformation shows a typical “target-like” appearance (arrows). Abdominal radiograph (**e**) of the same patient of 9**c**, **d**)—Retrospectively, the intraduodenal mass well-delineated by luminal air was already evident (arrows)

Gastric intussusception occurs secondary to a mobile gastric tumor [adenoma, GIST (Fig. 16), lipoma, hamartoma, polyp, adenocarcinoma] that prolapses into the duodenum [24].

Typical findings on UGIS include foreshortening and narrowing of the gastric antrum, converging or telescoping of mucosal folds in the antrum or duodenum, prepyloric collar-shaped outpouchings, and widening of the pyloric canal or duodenum, with an associated intraluminal filling defect in the duodenum which corresponds to the lead point (tumor).

CT shows and characterizes the leading point lesion and may demonstrate a bowel-within-bowel configuration, in which the layers of the bowel are duplicated forming concentric rings (when axial to the lumen) or a soft-tissue sausage-like image (when imaged longitudinally).

### Juvenile polyposis

Duodenal polyps are very rare findings. They most commonly are multiple, in the setting of polyposis syndromes such as Peutz-Jeghers Syndrome, Familial Polyposis, Gardner Syndrome, and Juvenile Polyposis [25].

Juvenile polyposis is most common in the colon, but occasionally involves the small bowel and duodenum [26]. These polyps contain cysts filled with mucin, so they won’t enhance after intravenous (iv) contrast administration.

The differential diagnosis of multiple polypoid lesions includes metastases, lymphoma, nodular lymphoid hyperplasia, and Kaposi sarcoma.

UGIS show radiolucent filling defects with mucosal origin (bowler hat sign). They have to be differentiated from pseudopolyps which are postinflammatory filling defects that occur in CD and ulcerative colitis, due to regeneration of nonulcerated tissue. A true polyp is round, regular, and well delineated, while an inflammatory pseudopolyp is usually y-shaped or filiform, irregular, and fuzzy with surrounding inflammation.

**Fig. 17:**
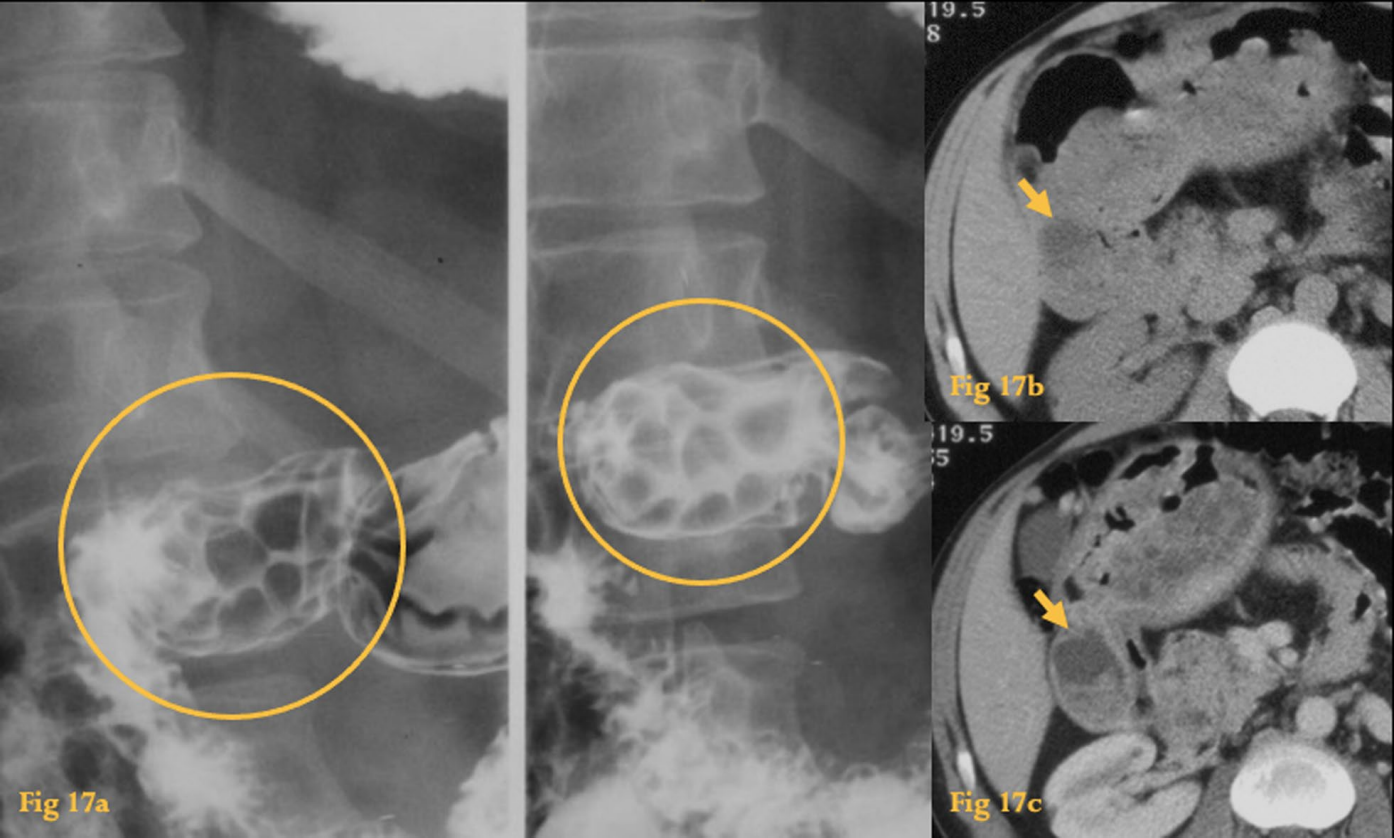
Juvenile polyposis. UGIS (**a**) with multiple filling defects at the duodenal bulb (circles). Unenhanced CT of another patient (**b**) shows a fluid density endoluminal lesion at the duodenal bulb (arrow). Enhanced CT of the same patient (**c**) shows several polyps at the duodenum and stomach. The biggest is located in the duodenal bulb and presents central fluid density with very little enhancement (arrow)

On CT, they appear as endoluminal lesions that may or may not enhance (Fig. 17).

### Kartagener syndrome—situs inversus

*Situs solitus (*normal situs) is inferred when the aortic arch, cardiac apex, and stomach bubble are located on the left. When these organs are on the right, *situs inversus* is present. So, *situs inversus* refers to an anatomic arrangement that is the mirror image of *situs solitus* [27].

It is found in 0.01% of the general population. 20% of patients with *situs inversus* also have Kartagener Syndrome (which includes male sterility, bronchiectasis, and sinusitis) [28].

**Fig. 18:**
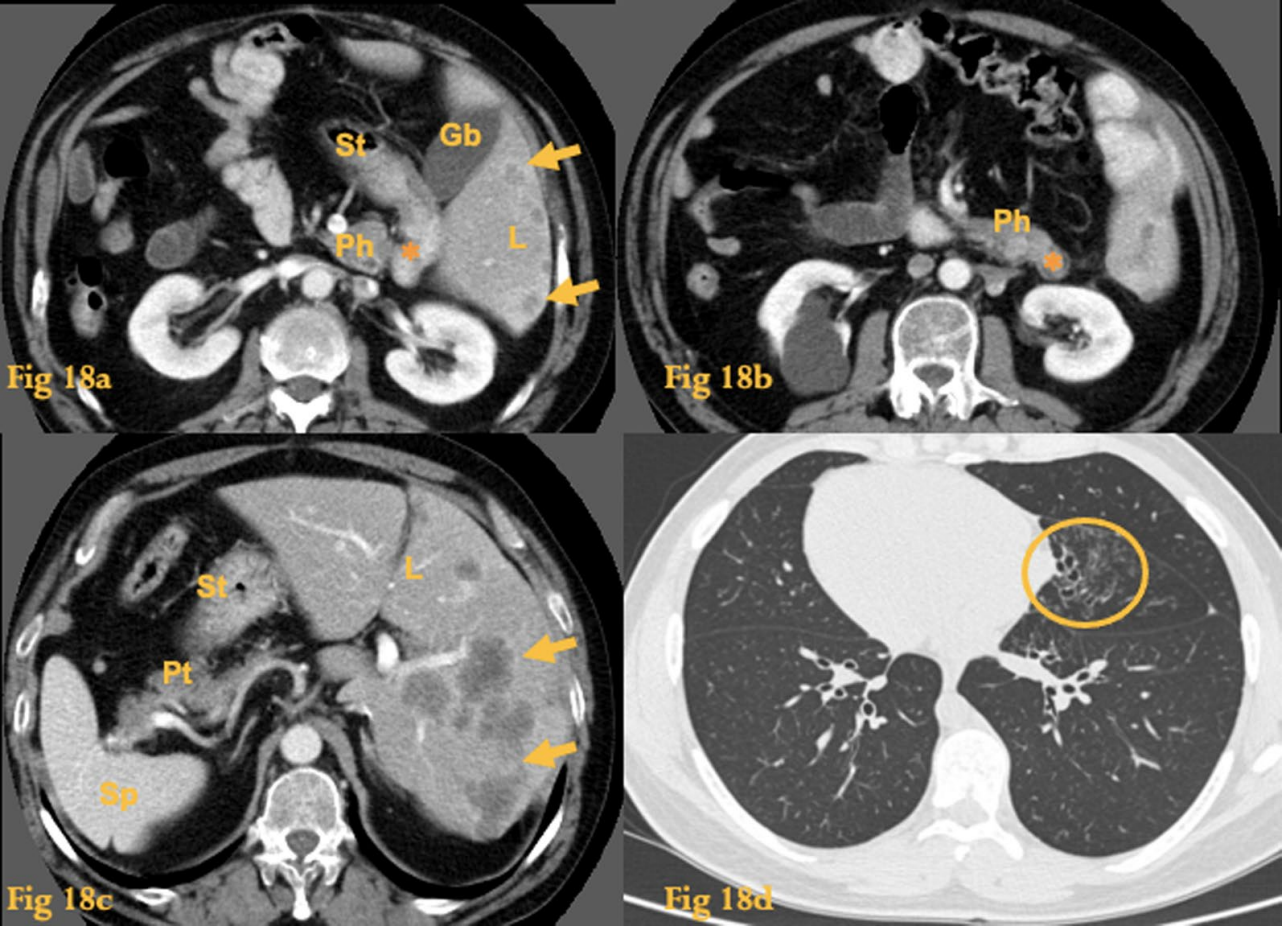
Kartagener syndrome. Abdominal CT of a patient with situs inversus. There are hypodense liver lesions (arrows) corresponding to metastases from colon cancer (**a**, **c**). The duodenum accompanies the mirror image of the other abdominal organs. Asterisk = descending duodenum. L = liver; S = spleen; St = stomach; Ph = head of the pancreas; Pt = tail of the pancreas; Gb = gallbladder. Thoracic CT of another patient (**d**) with situs inversus and Kartagener syndrome shows bronchiectasis in the middle lobe of the left lung (circle)

On CT, there is a mirror-image anatomy of the viscera (Fig. 18). The heart and great vessels are a mirror image of their normal anatomy; the left hemithorax contains a lung with 3 lobes, whereas the right hemithorax contains a lung with 2 lobes; the liver and gallbladder are on the left side, whereas the spleen and stomach are on the right side. The remaining internal structures, including the duodenum, are also a mirror image of the normal.

When there are no associated diseases, the only importance of recognizing the presence of this condition is due to the fact that an eventual surgical procedure can be done with an adequate surgical incision.

### Lipoma

Lipoma of the gastrointestinal tract is a submucosal soft-tissue tumor composed of adipocytes with an enveloping fibrous capsule [29].

Duodenal lipomas are usually solitary and can occur anywhere in the duodenum.

The majority of lipomas are found incidentally. They occasionally present as the leading point of an intussusception.

UGIS show smooth, oval, or spherical submucosal filling defect with very distinct margins and easily compressible on palpation during fluoroscopy.

**Fig. 19:**
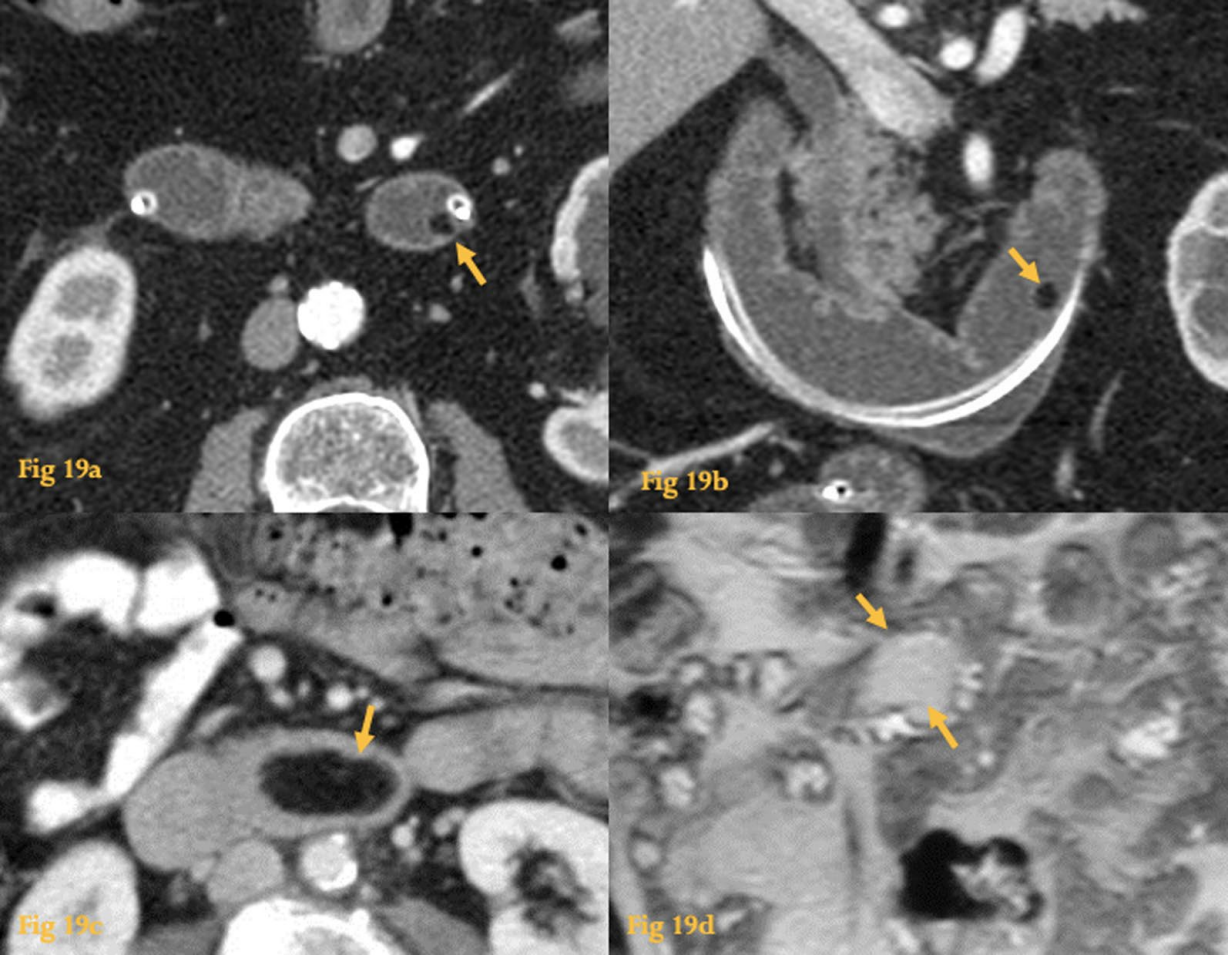
Lipoma. CT enteroclysis, axial (**a**) and coronal reformation (**b**) a small endoluminal nodule, with fat density is seen protruding of the wall at D4 (arrows). Abdominal CT of another patient (**c**) shows a duodenal mass with homogeneous fat attenuation in the horizontal duodenum (arrow). MR coronal HASTE image (**d**) shows the mass has high signal intensity (arrows), equal to the mesenteric fat

A definite diagnosis can be made by CT or MR by the demonstration of an endoluminal uniform fat-density nodule (Fig. 19).

Very rare complications such as ulceration and intussusception may, however, obscure the diagnosis.

### Lymphoma

Lymphoma of the gastrointestinal tract is most common at the distal ileum while the duodenum is the least frequent site [30].

Muscular wall replacement by tumor and destruction of the autonomic plexus causes typical aneurismal dilatation of the lumen (more frequent in the small bowell), so obstruction is less likely.

**Fig. 20:**
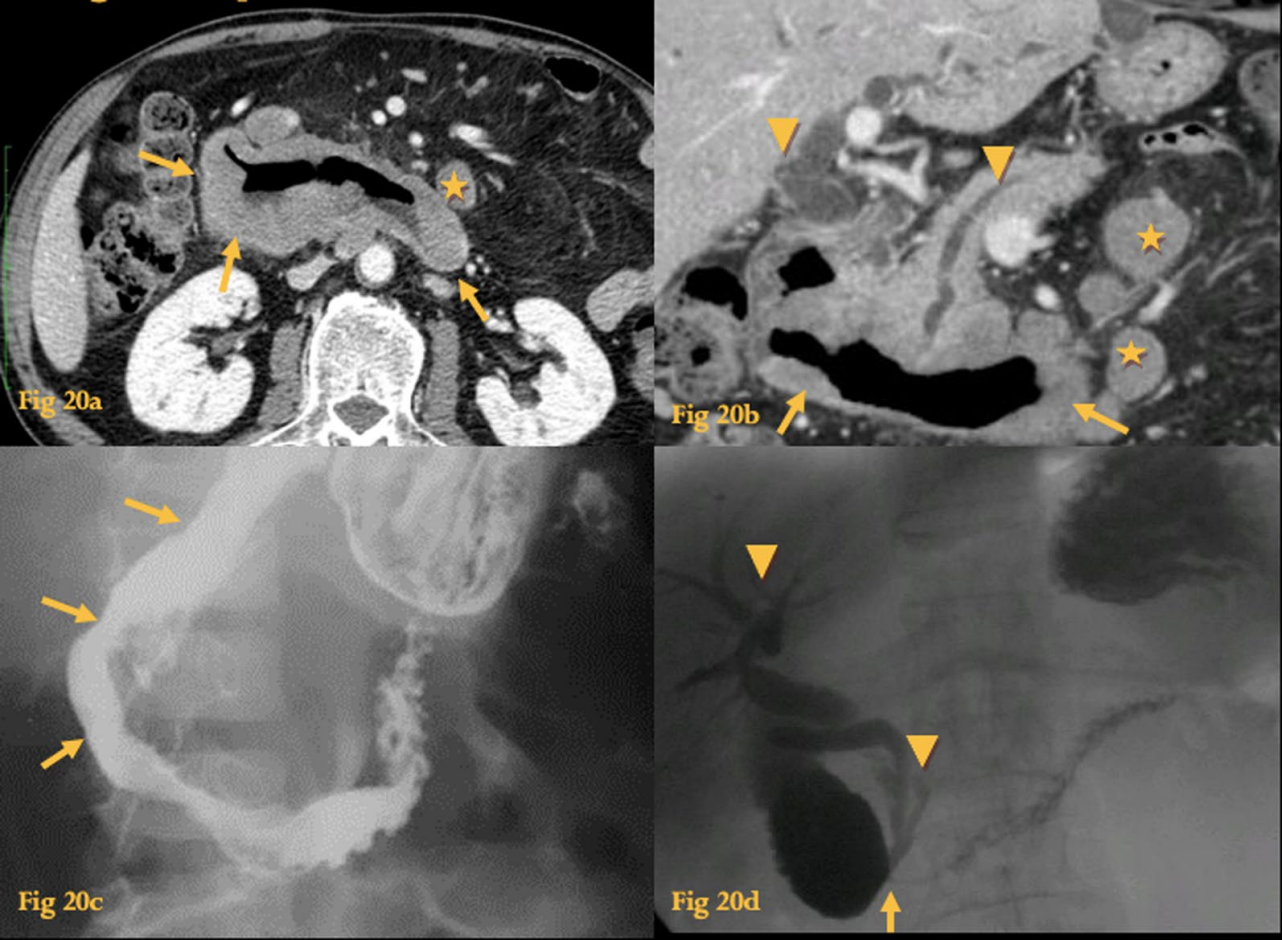
Lymphoma. Contrast-enhanced CT (**a**, **b**) shows homogeneous and asymmetric thickening of the duodenal wall with mild dilatation of the lumen (arrows); there is biliary and Wirsung duct dilatation (arrowheads); paraduodenal adenopathy is also evident (asterisks). UGIS of the same patient (**c**) shows loss of the normal mucosal pattern of folds at the descending duodenum (arrows). UGIS some months after the first UGIS, while on chemotherapy (**d**) shows abrupt luminal narrowing at the horizontal duodenum (arrow), with dilatation of the proximal duodenal segments. Because of increased pressure at the stenosis, there was contrast reflux to the biliary ducts and Wirsung duct (arrowheads)

On UGIS, thickened or effaced mucosal folds, with narrowed, normal, or dilated lumen is seen (Fig. 20c, d).

On CT, it manifests as a long segment of circumferential and asymmetric wall thickening that is of relatively homogenous tissue density and has little enhancement (Fig. 20a, b). It may also present with aneurysmal dilatation, an exophytic or polypoid mass or, rarely, obstruction. Bulky paraduodenal and retroperitoneal adenopathy is typically associated [25].

### Metastasis

Metastatic lesions of the small intestine are more frequent than primary tumors [31]. Duodenal metastases are most frequently located in the periampullary region, followed by the duodenal bulb. The most common primaries are breast, lung, and other GI tumors.

**Fig. 21:**
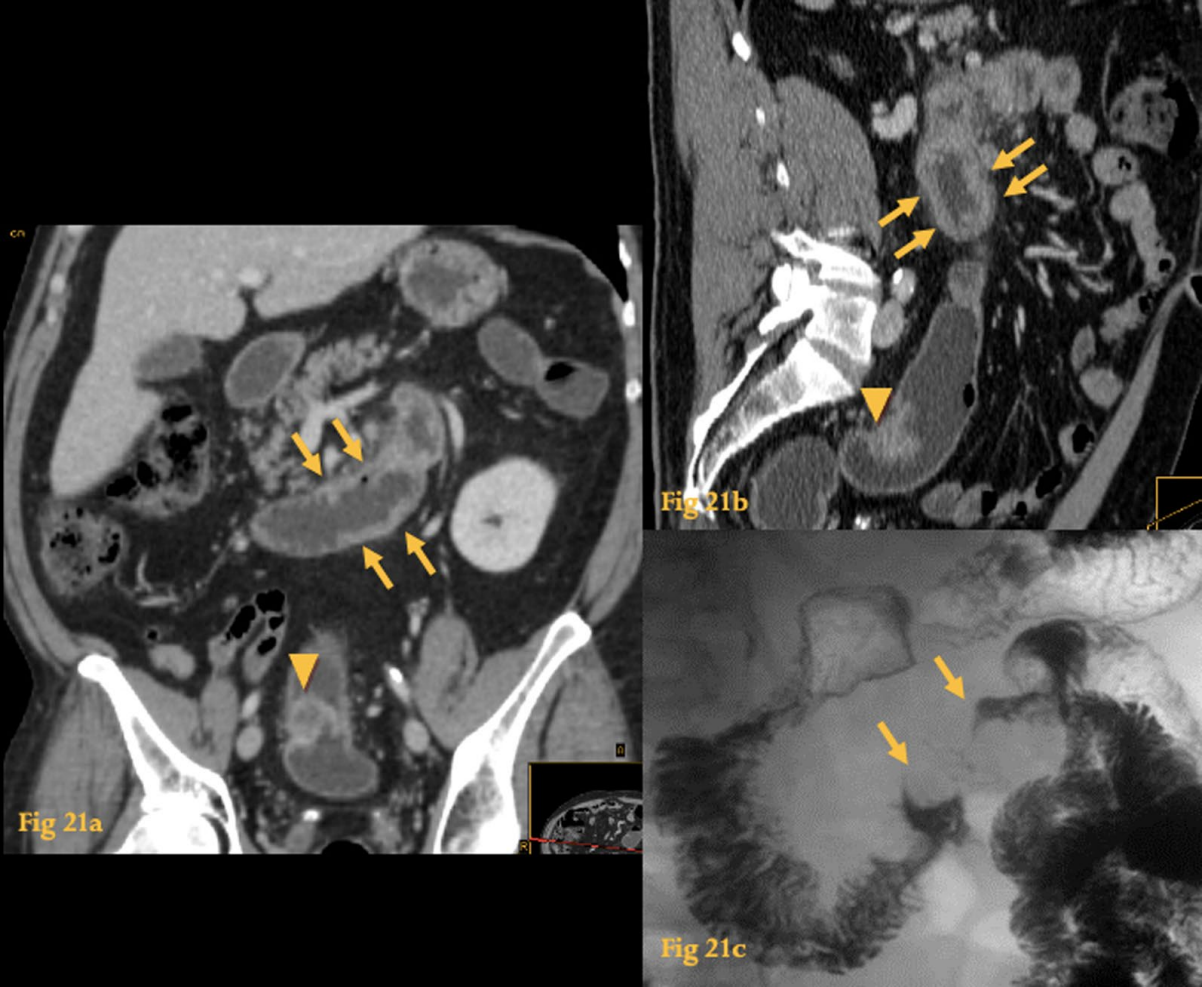
Metastasis. Abdominal and pelvic CT (**a**, **b**) of a patient with sigmoid colon cancer, performed to stage the disease, clearly demonstrated the sigmoid tumor (arrowheads) but failed to show distant metastases. However, MPR and retrospective analysis revealed a discrete circumferential thickening on D3 and D4 (arrows) which was not recognized initially. This duodenal lesion progressed to become an “apple core” lesion (arrows) causing obstructive symptoms, as evaluated by a barium study performed 2 months later (**c**)

Metastasis to the duodenum may occur in the wall or subserosa of the duodenum, but as they grow, they can extend into the lumen and present as intraluminal masses on UGIS (Fig. 21c), that may even ulcerate.

Although there are no specific imaging features of metastasis on CT, thickening of the bowel wall and folds in the involved segment may be seen (Fig. 21a, b) [25].

### Non-tropical sprue (celiac disease)

Celiac disease is a chronic autoimmune condition of gastrointestinal malabsorption that occurs in genetically susceptible individuals to gluten [32].

The bowel mucosa becomes flattened and villi disappear, while the submucosa, muscularis, and serosa remain normal. This destruction begins in the duodenum and progresses distally to the ileum over time.

These patients have an increased risk of lymphoma and gastrointestinal tract carcinoma.

The gold standard diagnostic test is a duodenal biopsy.

UGIS findings include duodenitis, small bowel dilatation, flocculation, effaced folds, decreased number of folds per inch of jejunum, and increased number of folds per inch in the ileum (≥ 5)—reversed jejunoileal fold pattern [25].

**Fig. 22:**
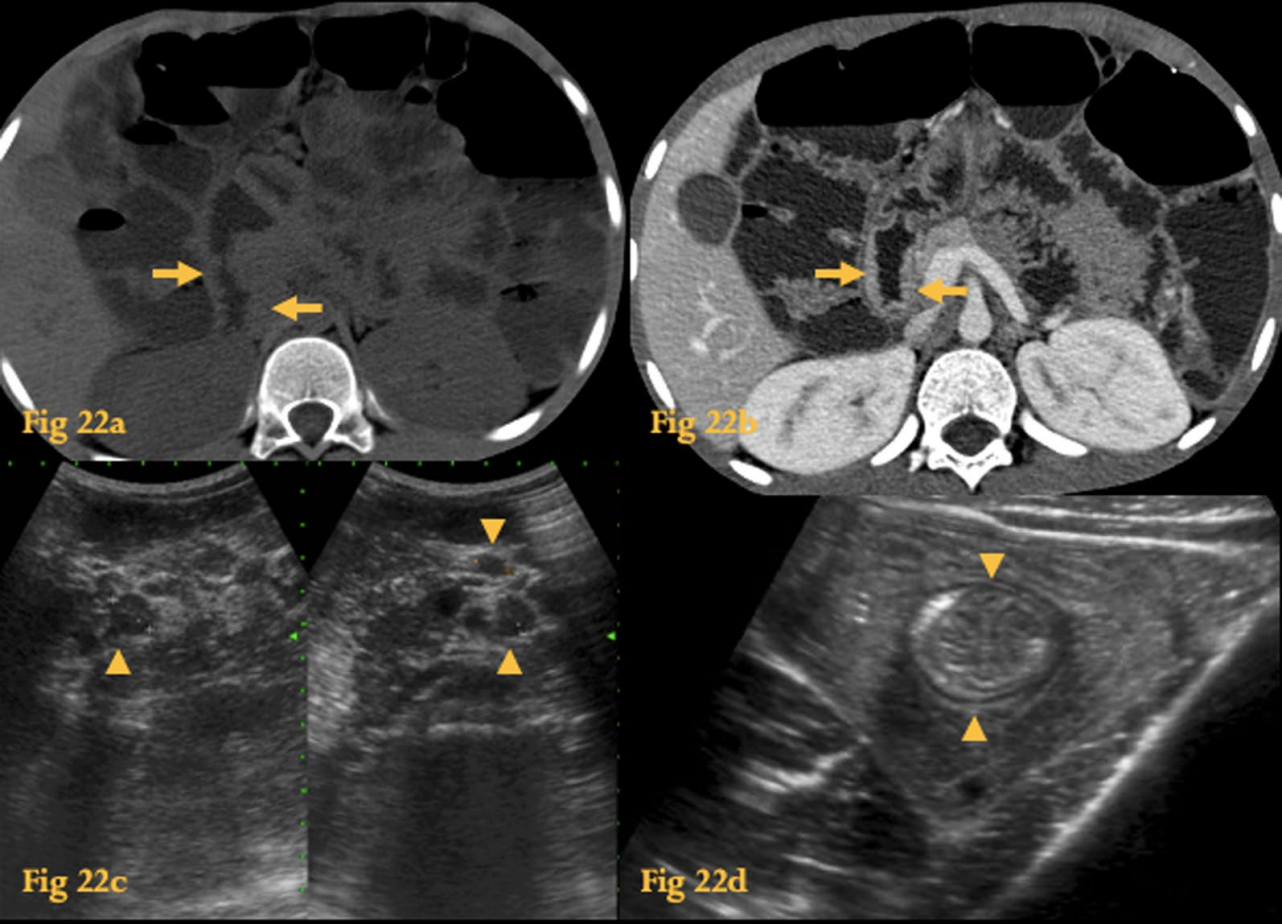
Celiac disease. Abdominal CT before (**a**) and after iv contrast administration (**b**) of a 14-year-old boy shows thickening and irregularity of the duodenal mucosa (arrows), with intense enhancement. Abdominal ultrasound of the same patient (**c**) detected several lymphadenopathies (arrowheads). Duodenal biopsy revealed disappearance of the “villi” associated with crypt hyperplasia, consistent with celiac disease. Abdominal ultrasound of another patient (**d**) who had three incidental transient small bowel intussusceptions (arrowhead), that were not present in a subsequent CT enterography a week later. Duodenal mucosa biopsy showed findings consistent with celiac disease

CT may show bowel wall thickening (Fig. 22), dilated small bowel, dilution of intraluminal contrast by fluid excess secretion, non-obstructing and transient intussusceptions, lymphadenopathy, and vascular engorgement.

### Neuroendocrine neoplasias (NEN)

NEN are rare neoplasms that arise from cells of the endocrine and nervous systems. These tumors originate within the pancreas or from similar neuroendocrine cells outside of the pancreas.

They are classified as functioning or non-functioning (if they secrete hormones or not); and they can be benign or malignant (60–92%).

Insulinomas and gastrinomas are the most common of these rare tumors.

**Fig. 23:**
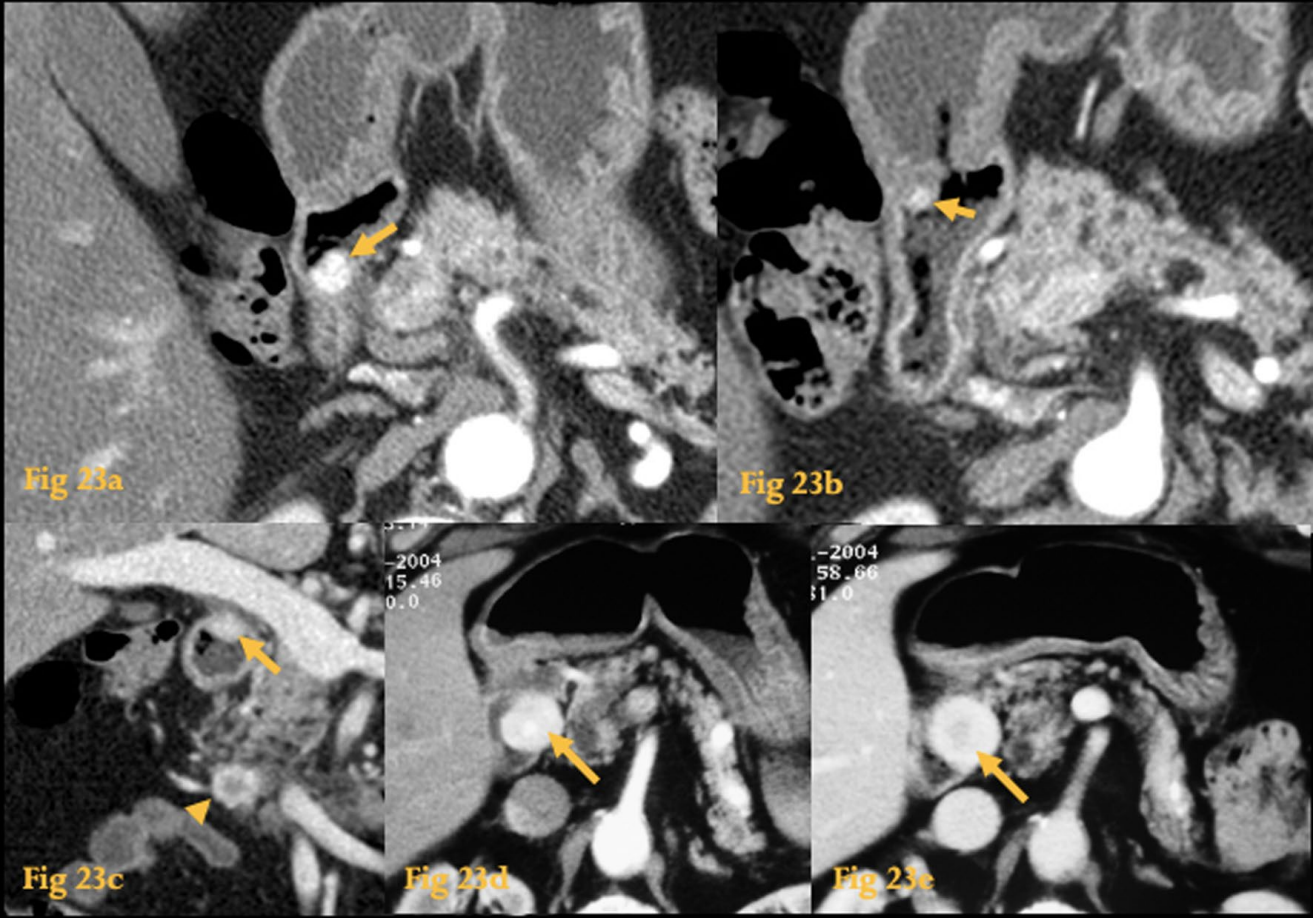
Neuroendocrine tumor. Arterial phase CT (**a**, **b**) of a patient with type I multiple endocrine neoplasia, with hypergastrinemia, shows two small hypervascular nodular lesions on the wall of D2 and D1 (arrows). Coronal reformation (**c**) better shows the duodenal bulb lesion (arrow) and another similar lesion at the pancreatic head (arrowhead). Histopathological study following pancreatoduodenectomy revealed duodenal and pancreatic neuroendocrine tumors (gastrinomas). Abdominal CT of another patient shows a mass (arrows) in the medial wall of D2 with intense contrast enhancement in the arterial phase (**d**) and venous phase (**e**) of the dynamic study. The high spatial resolution provided by CT clearly shows that the lesion is independent from the pancreas

While insulinomas are almost always found in the pancreas, gastrinomas are found within the gastrinoma triangle in 90% of cases, an anatomical area which includes the head of the pancreas and D1 and D2 (Fig. 23).

NEN grade 1–2 are usually hyperdense, but NEN grade 3, as a rule, have a heterogeneous structure with a pronounced hypodense component and liver metastases. The higher the NEN grade, the less intense contrast enhancement, and the tumor itself is more heterogeneous. Most often NEN grade 2 mimic GIST. In the absence of intense contrast enhancement, a large tumor can mimic adenocarcinoma.

On CT, they manifest as a small nodule in an appropriate location, with enhancement to a greater degree than the pancreas during arterial and portal venous phases. They can be multiple in 10%. They may mimic arterial structures when in a peripancreatic location [2]. Water should be used as a negative oral contrast to increase the conspicuity of these tumors when located in the duodenum.

### Occlusion (paraduodenal internal hernia)

Internal hernias (IH) are defined as the protrusion of viscera through a normal or abnormal peritoneal or mesenteric aperture within the peritoneal cavity. Paraduodenal hernias are classically regarded as the most prevalent IH (53% of the cases), with 75% of them occurring on the left.

**Fig. 24:**
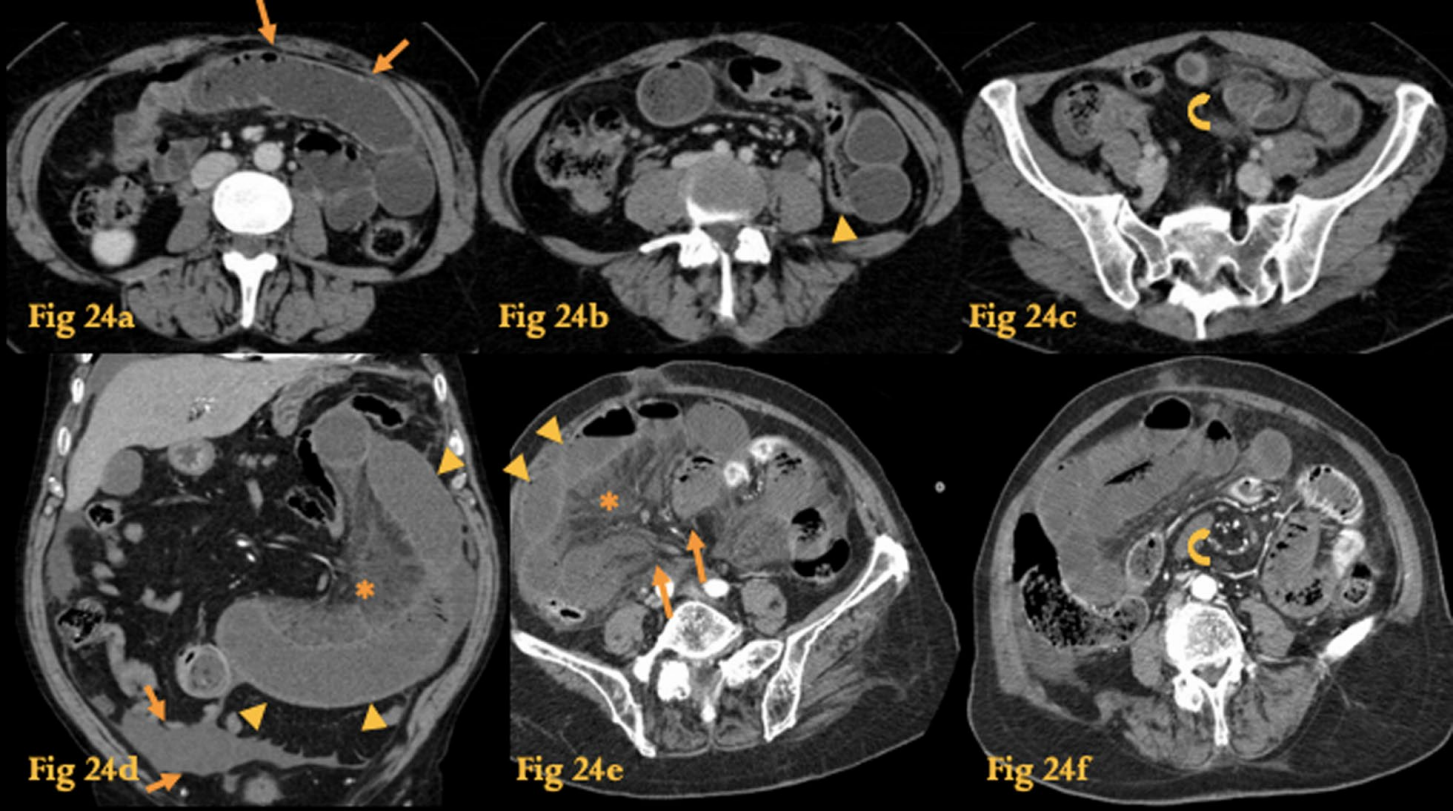
Internal hernia. Abdominal CT of a patient with a left paraduodenal internal hernia there is no fat between the fluid-filled dilated small bowel loop and the anterior abdominal wall (arrows on **a**). The small bowel loops are seen lateral to the descending colon with medial deviation of the colon (arrowhead on **b**). There is twisting of the bowel loops and mesentery—whirl sign (curved arrow on **c**). Another patient with CT findings of a right paraduodenal internal hernia fluid-filled dilated loop of small bowel with a U/C-shape configuration which represents a closed loop obstruction (**d**). Both ends of a fluid-filled distended closed-loop taper fusiformly, in a similar way to a beak—the beak sign (arrows on **e**). There is also twisting of the mesenteric vessels at the mesenteric root—whirl sign (curved arrow on **f**). The fact that there is diminished enhancement of the bowel wall (arrowheads), densification of the mesentery (asterisk) and free fluid (arrows) suggests strangulation (**d**)

Left paraduodenal IH on the left develops through the fossa of Landzert located at the duodenojejunal junction [33]. Right paraduodenal IH involves the fossa of Waldeyer, which is located immediately behind the superior mesenteric artery (SMA) and inferior to D3 and is usually associated with nonrotation (Fig. 24d–f).

On UGIS crowding of small-bowel loops in the hernial sac, apparent encapsulation of dilated bowel loops in an abnormal location, segmental distension with stasis, and reversed peristalsis may be seen.

CT of left paraduodenal IH shows a saclike mass of dilated bowel left to the ligament of Treitz and between the pancreas and the stomach that displaces the transverse colon and duodenal flexure inferiorly (Fig. 24a–c). The mesenteric vessels are crowded, engorged, and stretched at the entrance of the hernial sac (the whirl sign). The loops of the hernial sac classically “adhere” to the abdominal wall without overlying omental fat. Typical findings of closed-loop obstruction are also usually found: radial distribution of the loops, an U/C configuration of the bowel loop, and the beak sign [34].

### Paraduodenal pancreatitis

Paraduodenal pancreatitis is an uncommon form of segmental chronic pancreatitis that affects the pancreaticoduodenal groove near the minor papilla. It no longer should be called groove pancreatitis.

The hallmark of the disease is the presence of scar tissue with fibrosis in the pancreaticoduodenal groove, which may lead to duodenal stenosis [35].

**Fig. 25:**
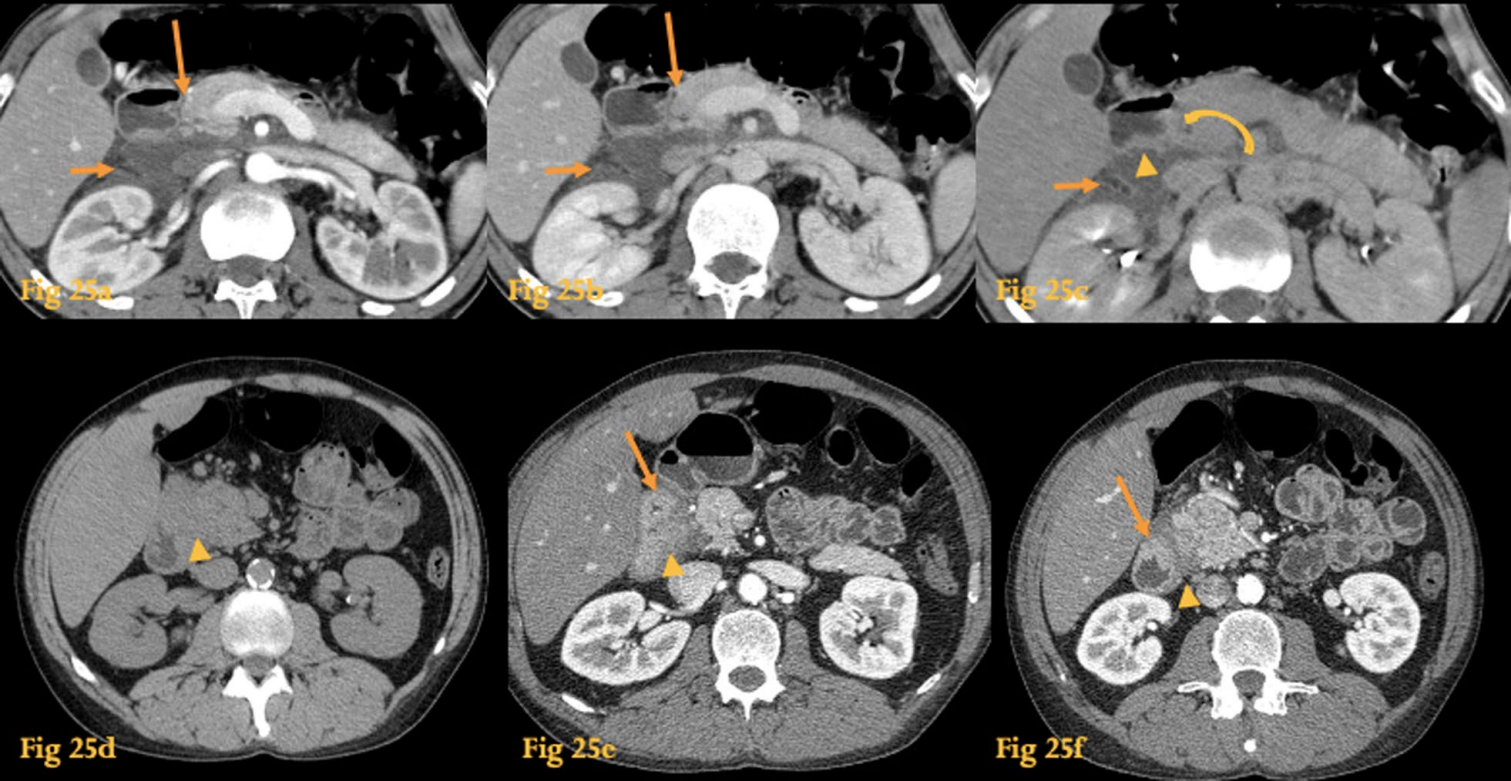
Paraduodenal pancreatitis. Abdominal CT of a patient with clinical and laboratory findings suggestive of pancreatitis (**a**–**c**). The pancreas has normal volume, morphology, and texture. There is retroperitoneal fluid in the right anterior pararenal space (small arrows) and in the pancreatoduodenal groove (big arrows). The posterior wall of the descending duodenum is slightly thickened (arrowhead) and the pancreatoduodenal groove reveals discrete late enhancement (curved arrow on **c**), suggestive of fibrotic changes. Abdominal CT of another patient with paraduodenal pancreatitis (**d**–**f**) shows an ill-defined soft-tissue mass within the groove (arrowheads) and descending duodenal medial wall thickening (arrows)

On CT, there’s ill-defined sheet-like soft-tissue attenuation material between the pancreatic head and the descending duodenum that may show late enhancement (Fig. 25). Duodenal wall thickening and stenosis may be seen. Small cystic lesions in the thickened duodenal wall may be present [36] (*see letter C for cystic dystrophy*).

Differentiation from duodenal or periampullary tumors is difficult with imaging alone, although the presence of cystic lesions within the mass would favor a diagnosis of paraduodenal pancreatitis. Sometimes, the two entities may coexist, since the inflammation is cause and/or consequence of cancer.

### Annular pancreas

Annular pancreas is an uncommon morphologic congenital anomaly that occurs following failure of the pancreatic ventral bud to rotate with the duodenum, resulting in the envelopment of the duodenum [20].

This condition can manifest in the 1st decade of life with duodenal stenosis, although the diagnosis is difficult at this stage due to lack of intra-abdominal fat in children and the narrow width of the pancreatic band.

Half of the cases occur in adult life, with 66% of them remaining asymptomatic. When symptomatic, the presentation is usually with abdominal pain, postprandial fullness, vomiting, post-bulbar peptic ulceration, pancreatitis, or biliary obstruction.

UGIS show an eccentric or concentric narrowing of D2.

**Fig. 26:**
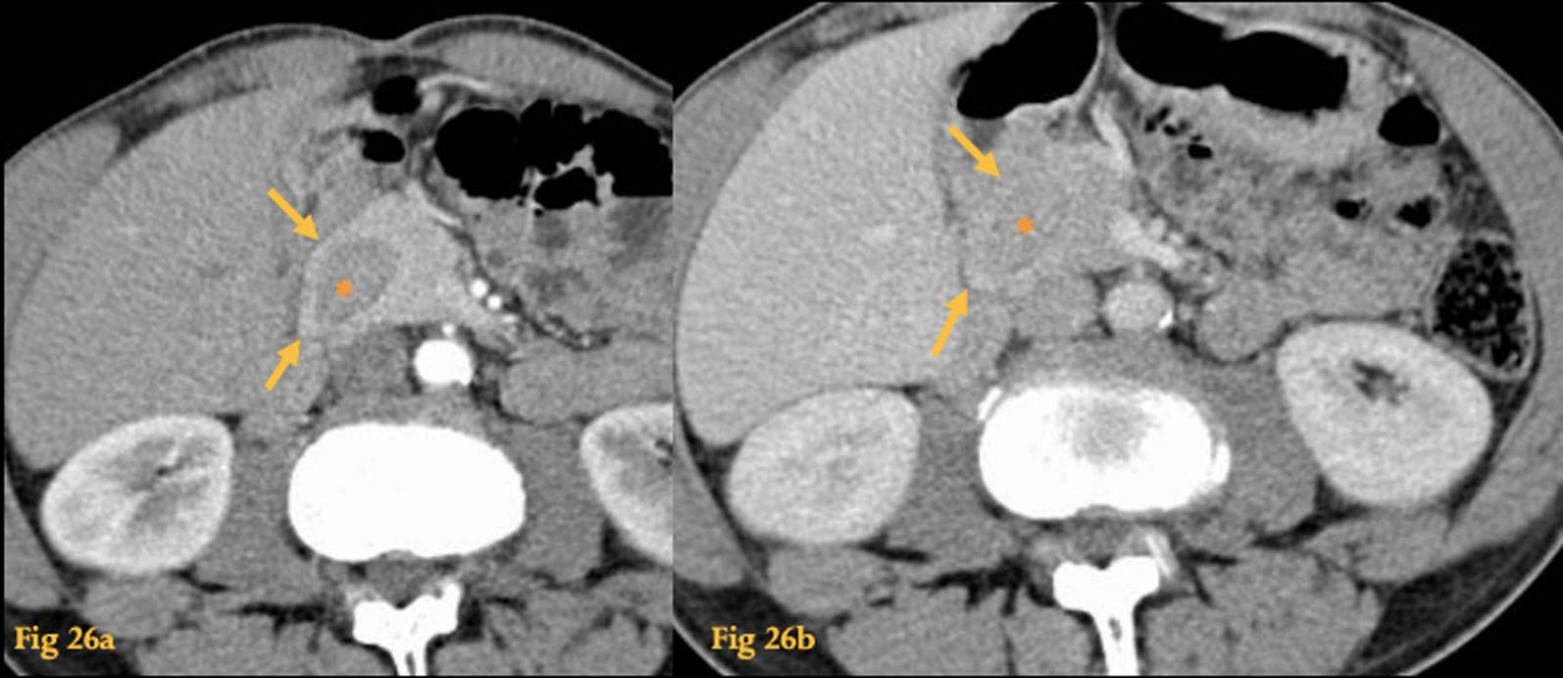
Annular pancreas. Abdominal CT of an adult patient shows pancreatic tissue completely surrounding D2 (asterisk), in an annular fashion (arrows), with enhancement equal to the pancreas on both the arterial (**a**) and portal venous (**b**) phases

On CT, pancreatic tissue completely or incompletely surrounds the descending duodenum (Fig. 26), sometimes with associated duodenal narrowing and dilatation of proximal duodenum. Negative oral contrast material and imaging in the arterial phase to view the enhancing pancreatic tissue may help achieve the diagnosis.

ERCP or MRCP allows for accurate delineation of the annular duct anatomy that usually joins the Wirsung or Santorini ducts.

### Quiescent and active Crohn’s disease

Crohn’s disease is the most common inflammatory condition of the small bowel, with an incidence ranging from 5 to 60% at the duodenum. It usually involves the D1 and D2, almost always associated with continuous involvement of the stomach. The first evidence of the disease tends to manifest as ulcer or stricture formation, while late involvement typically occurs as fistulous tracts from an adjacent diseased loop of small bowel or colon.

**Fig. 27:**
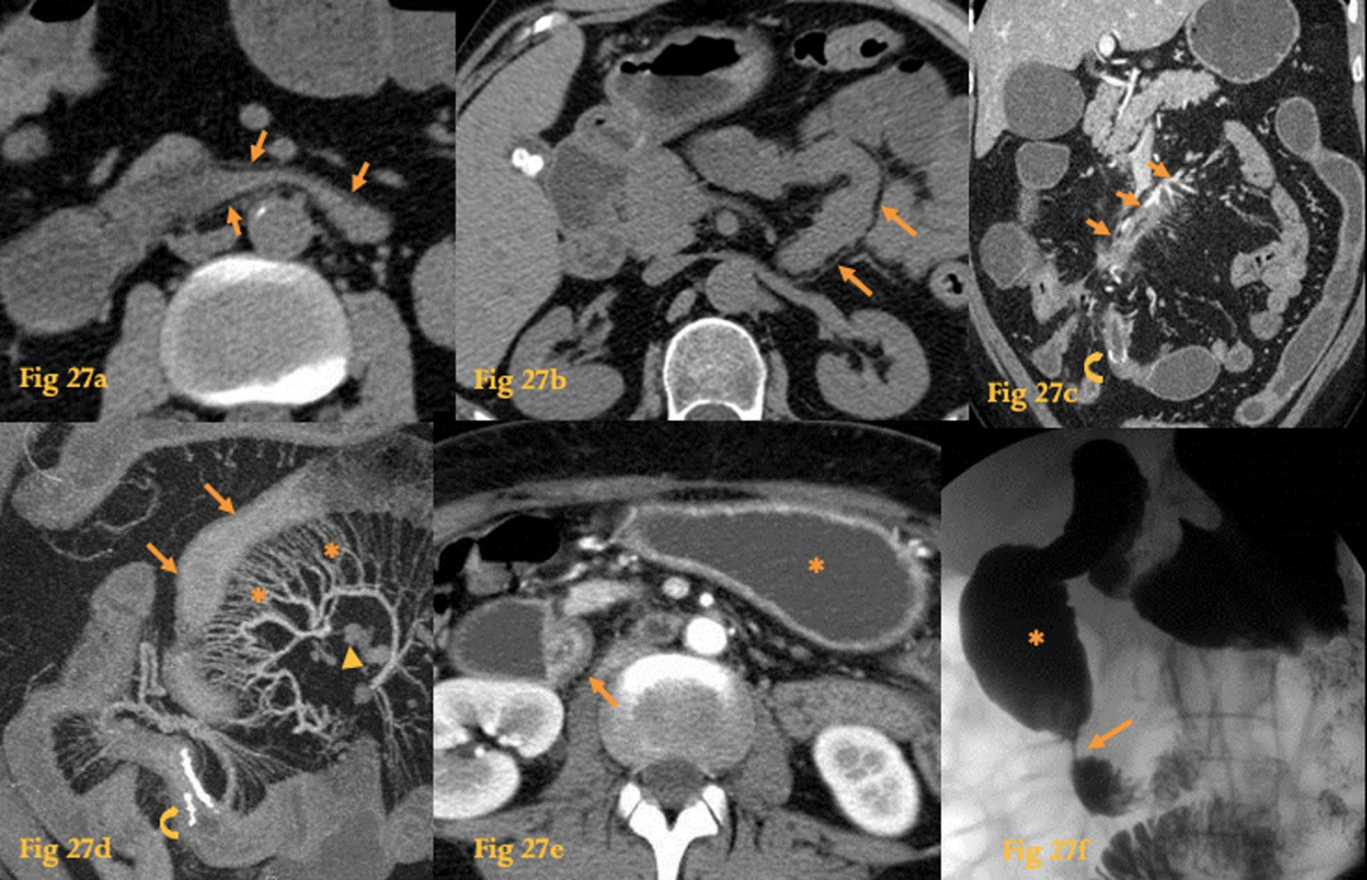
Chron’s Disease. Plain abdominal CT shows the fat halo sign (arrows) in the wall of the D3 (**a**) and D4 (**b**), in a patient with quiescent Crohn’s disease. CT enterography (**c**) of a CD patient with active disease who previously had a segmental enterectomy—note the surgical clips at the ileal-ileal anastomosis (curved arrows). There is a fistula between the thickened ileal segment near the anastomosis and the duodenum. MIP coronal reformation (**d**) shows mesenteric engorgement (asterisks) and adenopathy (arrowhead) near a thickened bowel wall segment (arrows). Abdominal CT of another patient with active disease (**e**) shows gastric outlet obstruction (asterisk) and a stricture in D2 that shows the target sign (arrow). Barium study of the same patient (**f**) shows the short stricture (arrow) and dilatation of the proximal duodenum (asterisk)

The UGIS main advantage over CT is the ability to depict aphthous ulcers in the active phase [25]. Other possible findings of CD are luminal narrowing (Fig. 27f), thickened folds, and fistulous tracts.

CT of active disease may reveal hyperenhancing duodenal wall thickening, mesenteric edema, prominent mesenteric vessels (comb sign), and lymphadenopathy (Fig. 27d), as well as possible fistulas or abscesses [2]. A typical finding is a target sign, which corresponds to the enhanced mucosa and serosa with a hypodense and edematous submucosa between them. Homogeneous enhancement of the duodenal wall suggests quiescent disease, although a fat halo sign, which corresponds to a fat attenuation layer between an inner and an outer layer of soft-tissue attenuation, resulting from fatty infiltration of the submucosa, may also be seen at this stage (Fig. 27a, b).

MR enterography can demonstrate active small-bowel inflammation and complications such as bowel obstruction, penetrating disease, and abscess formation, with multiplanar capability, and functional studies (motility, perfusion, diffusion), without the use of ionizing radiation making this method very suitable for a young population with CD that will likely undergo many imaging examinations throughout their lives [37].

### Rupture

Duodenal perforation can result from peptic ulcers, perforated tumors, iatrogenic trauma (ERCP), or non-iatrogenic trauma [motor vehicle accidents, ingested foreign objects].

Duodenal perforation associated with peptic disease is commonly found in the bulb, whereas D2 and D3 are the common sites of perforation caused by blunt trauma (*see letter T for Trauma and letter U for Ulcers*).

Iatrogenic duodenal perforation is a complication of endoscopy or ERCP sphincterotomy and is usually suspected at the time of the examination.

**Fig. 28:**
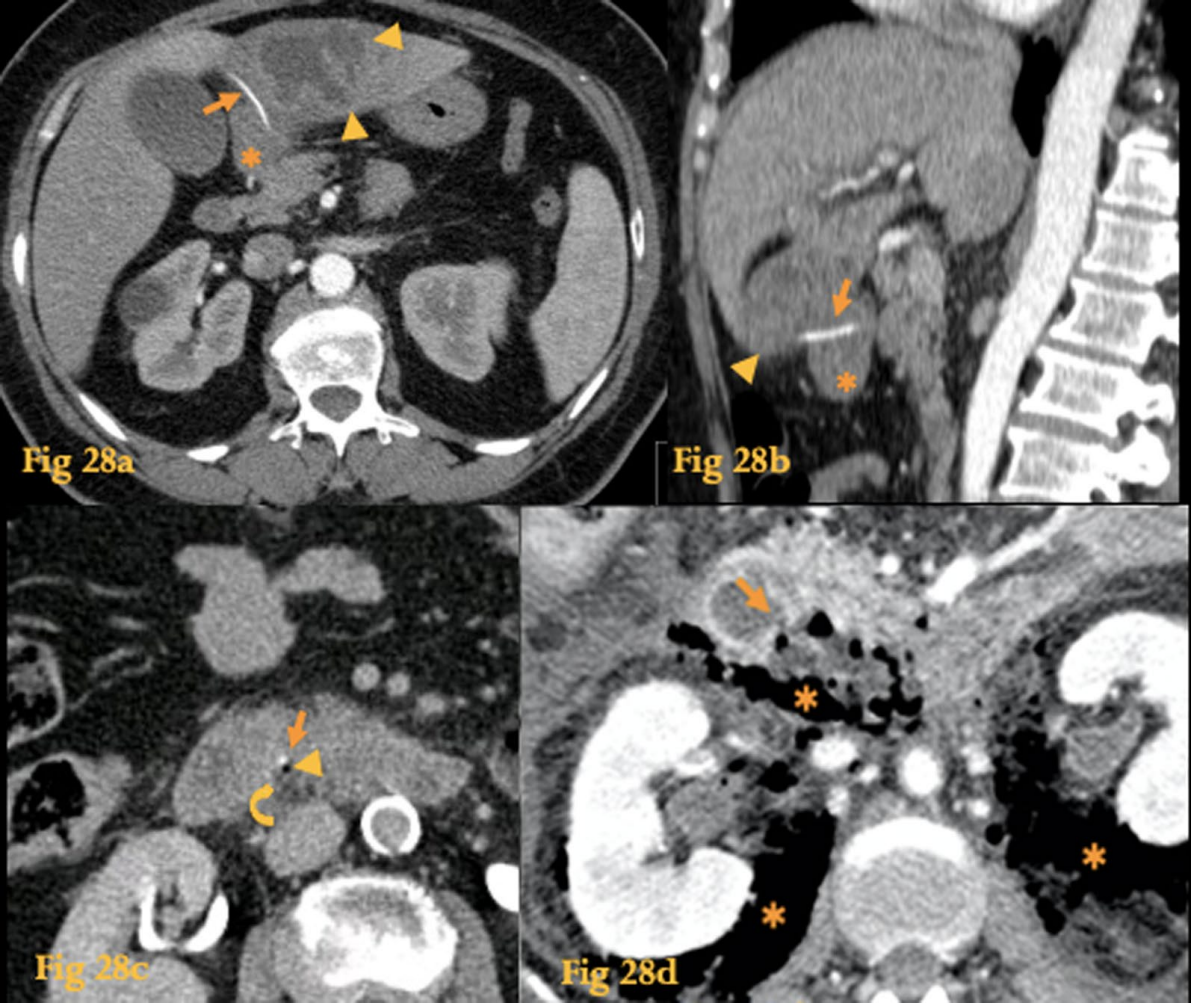
Rupture. Abdominal CT (**a**, **b**) of a patient who had ingested a fishbone (arrow). It perforated the duodenal bulb (asterisk) and lodged on the left lobe of the liver. Note the adjacent liver abscess (arrowheads) that formed as a result. CT of another patient (**c**) shows an endoluminal calcic density structure—fishbone (arrow) at D3, with a small amount of adjacent extraluminal gas (arrowhead) and fat stranding (curved arrow), indicative of retroperitoneal rupture. CT of yet another patient who had underwent ERCP (**d**) shows a discontinuity of the posterior wall of D3 (arrow) and extensive pneumoretroperitoneum (asterisk)

Typical findings on CT (Fig. 28) include intraperitoneal or retroperitoneal air and fluid adjacent to the duodenum, with or without evidence of bowel wall discontinuity [38]. If free air is found in the lesser sac (intraperitoneal), the likely site of perforation is the posterior wall of the stomach or duodenal bulb. Extraluminal free air in the right anterior pararenal space is a reliable CT finding for duodenal perforation in segments beyond the bulbar segment, which are retroperitoneal [39]. Indirect findings such as bowel wall thickening, abnormal bowel wall enhancement, abscess, and inflammatory changes adjacent to the bowel may be associated.

### Superior mesenteric artery syndrome

**Fig. 29:**
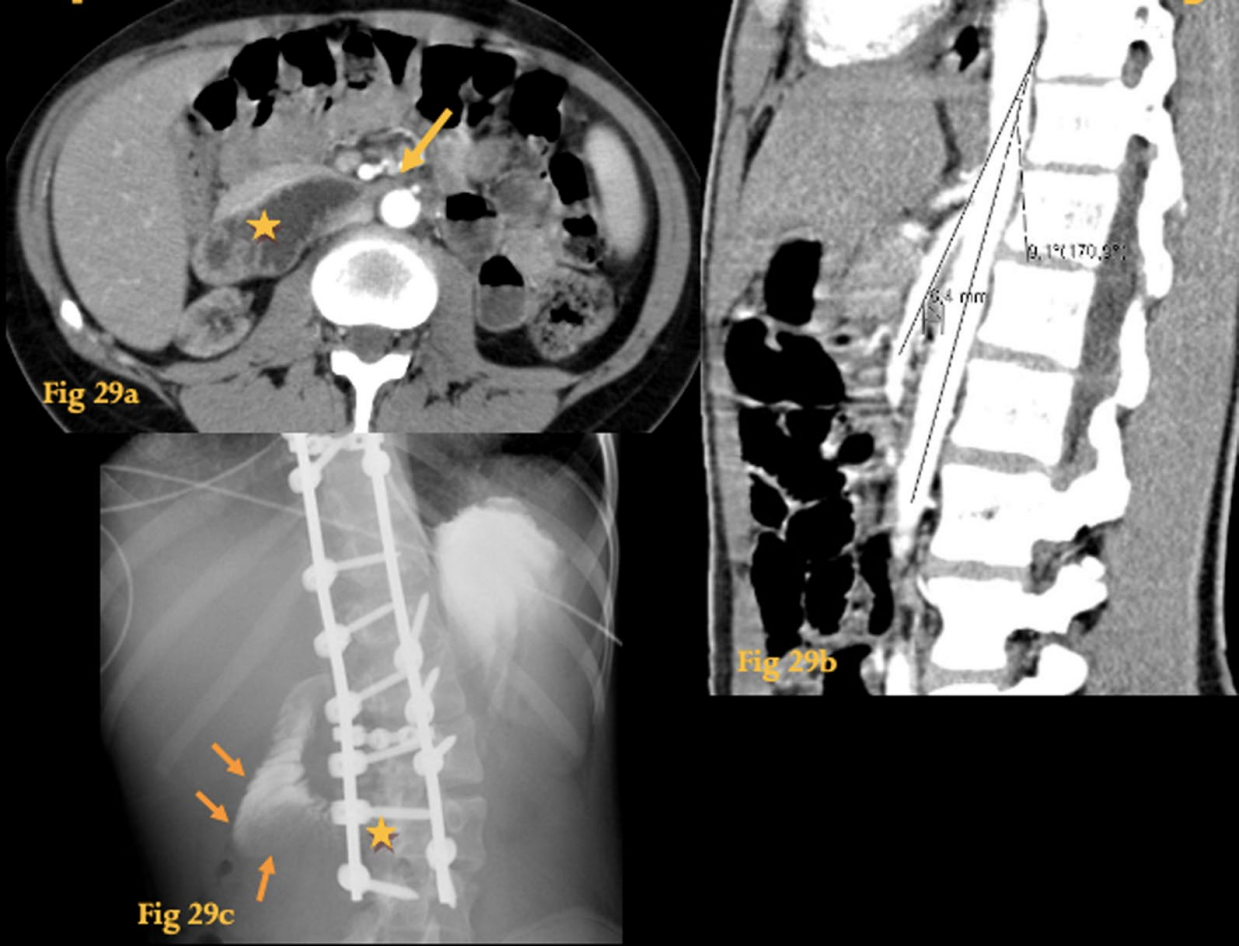
Superior mesenteric artery syndrome. Arterial phase of an abdominal CT study of a female adolescent (**a**) shows dilatation of the duodenum (asterisk) upstream to the point where it crosses the SMA and the aorta (arrow). Note paucity of abdominal fat. Oblique sagittal reformation of another patient (**b**) shows reduction of the normal aortomesenteric angle (9º) and aortomesenteric distance (6 mm). Fluoroscopic series of a 10-year-old girl after surgical correction of kyphosis **c** shows obstruction at D3 with delayed passage of contrast through the stenosis (asterisk) and proximal dilatation of D2(arrows). This was due to SMA syndrome caused by lengthening of the vertebral column

Superior mesenteric artery syndrome is an unusual cause of high intestinal obstruction, caused by extrinsic compression ofD3, due to narrowing of the normal angle between the aorta and the SMA (Fig. 29).

Patients are usually young females, with conditions associated with rapid and severe weight loss, resulting in a loss of retroperitoneal fat (AIDS, anorexia nervosa, other causes of cachexia).

On UGIS during a symptomatic episode, there is strong to-and-fro peristalsis and stomach and duodenal dilatation proximal to a vertical extrinsic impression at the level of D3. Relief of obstruction with postural change may be seen.

CT angiography (or MR angiography) allows measurement of the distance between the aorta and the SMA (Fig. 29b). Normally, the aortomesenteric angle and aortomesenteric distance are 28–65° and 10–34 mm, respectively. In SMA syndrome, both parameters are reduced, with values of 6° to 15° and 2 to 8 mm, respectively [40].

### Accessory spleen

Accessory spleens occur in 10–16% of normal individuals. They may be single or multiple and are most frequently found near the splenic hilum. Occasionally they can be found in the suspensory ligaments of the spleen or the pancreatic tail. Rarely they can occur elsewhere in the abdomen, like near the duodenum [2].

In most patients, they are incidental findings of no clinical significance. Sometimes it is important to identify accessory spleens when they are confused with a mass of another type, particularly when they occur in an atypical location.

**Fig. 30:**
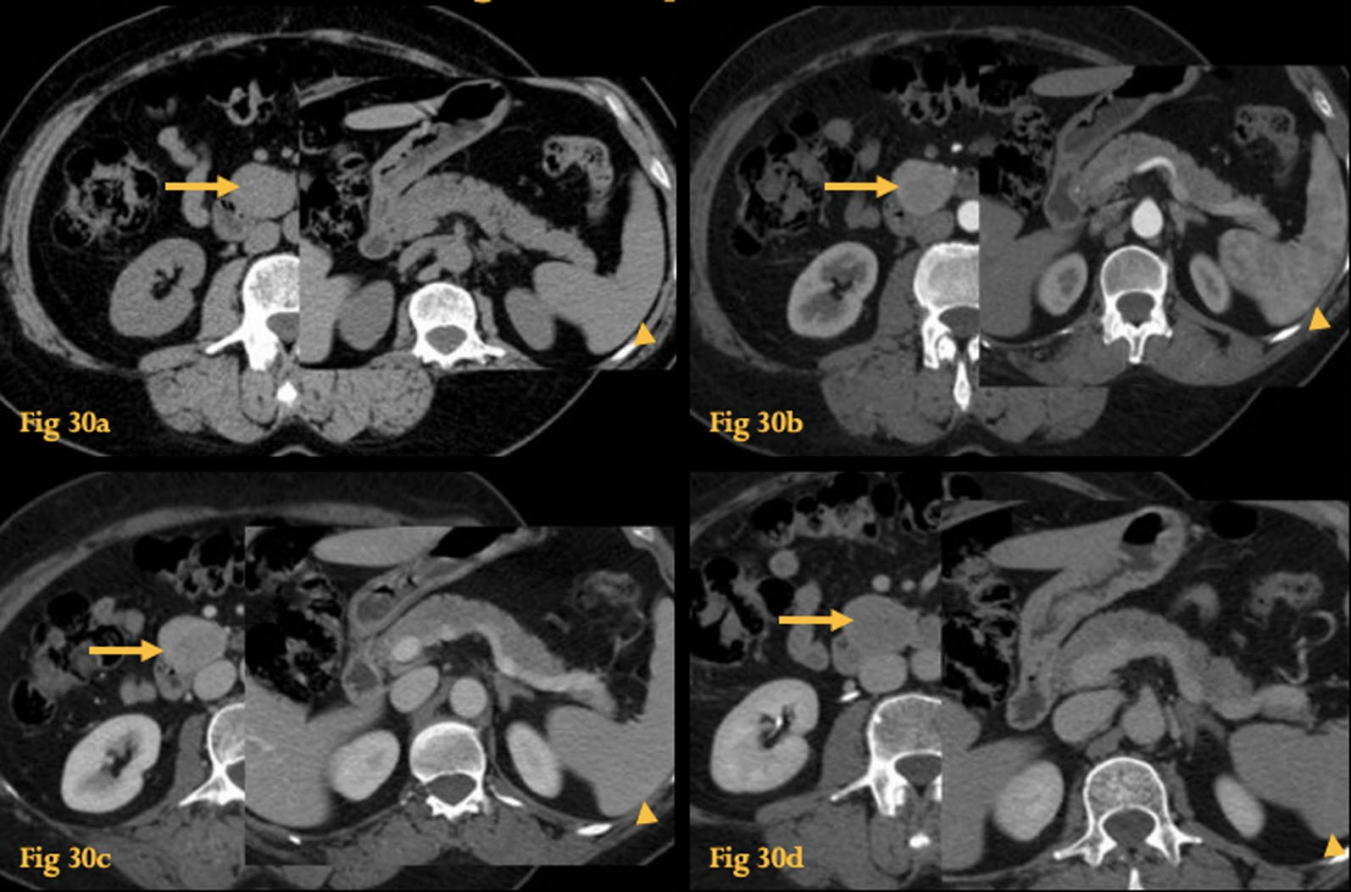
Accessory Spleen. Unenhanced (**a**), arterial phase (**b**), portal venous phase (**c**), and late phase acquisitions (**d**) CT show fortuitus finding of a peri-duodenal solid lesion, between the uncinate process and the third portion of the duodenum (arrows), with a smooth, round shape, and a pattern of enhancement similar to the normal spleen (arrowheads) on all phases. Histopathological study of the resected surgical specimen revealed splenic tissue

On CT and MR, they appear as round or ovoid masses, of regular margins, from 1 to 5 cm in size, with the same pattern of contrast enhancement/ dynamic behavior of the normal spleen [25] (Fig. 30).

Technetium sulfur colloid radionuclide scans can be used to confirm the diagnosis in problematic cases.

### Trauma

**Fig. 31:**
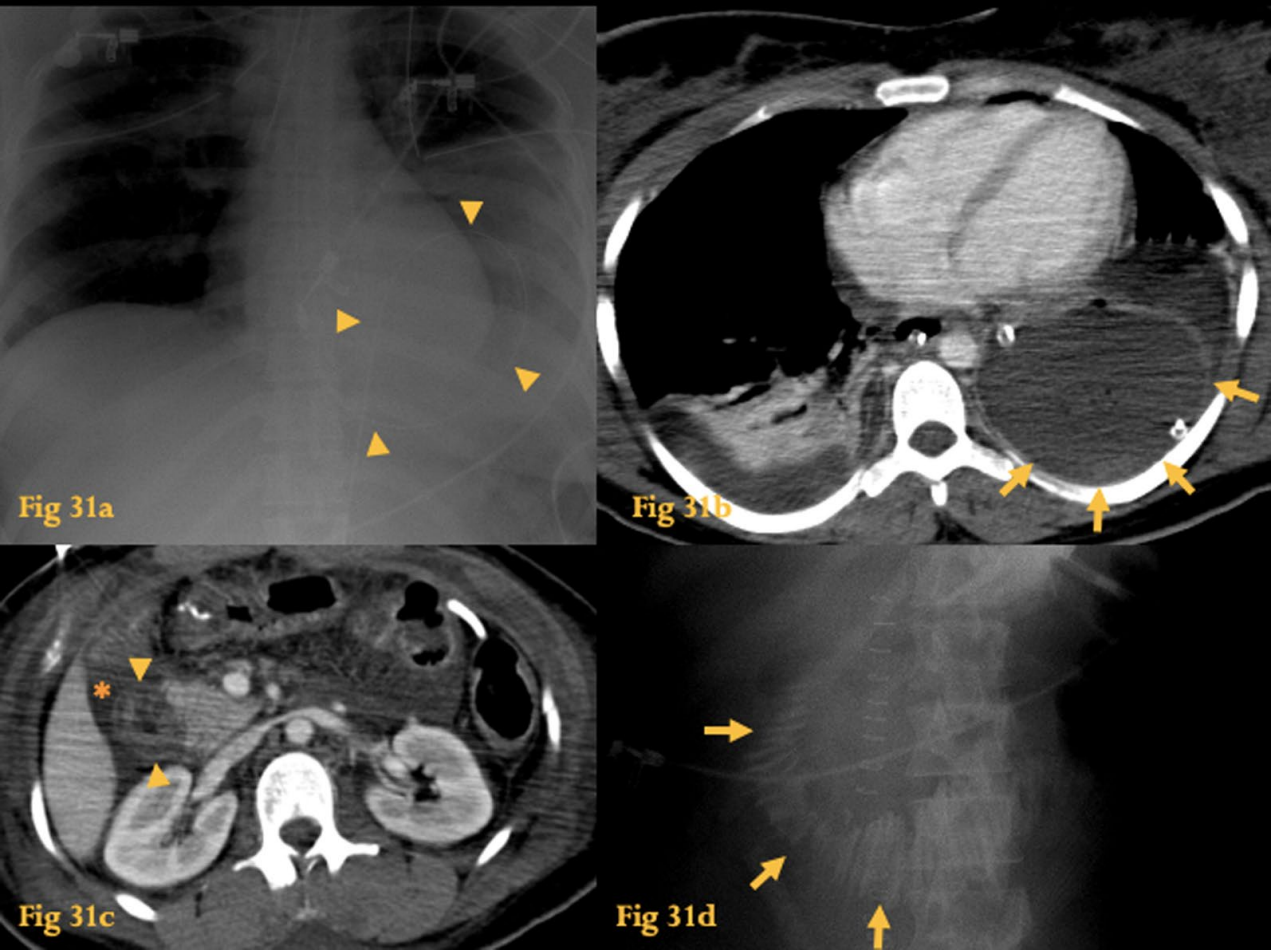
Trauma. Chest radiograph (**a**) of a car accident victim shows a gastric tube with a thoracic loop (arrowheads). Contrast-enhanced CT of the same patient (**b**) confirms traumatic diaphragmatic hernia; note the absence of interposition of lungs between the stomach and the chest wall—the so-called “dependent viscera sign” (arrows). Abdominal CT (**c**) shows that D2 is surrounded by fluid (asterisk) and its wall presents the target sign (arrowheads). The upper GI study (**d**) of the same patient revealed uniform, regular, fold thickening at D2 and D3, similar to a stack of coins (arrows), representing intramural edema/hematoma

Duodenal trauma may result from penetrating or blunt injury (for example in rapid deceleration car accidents—Fig. 31). During blunt trauma, the duodenum is compressed against the spine, causing contusion or transection.

Since most of the duodenum is retroperitoneal, peritoneal signs are usually absent, so CT plays a pivotal role in deciding patient treatment [39]. While duodenal contusion is usually managed conservatively, traumatic duodenal perforation requires emergency surgery [38].

On UGIS, intramural hemotoma may manifest as regular fold thickening, resembling a stack of coins (Fig. 31d).

In acute bowel injury, a target sign is seen on CT, composed of inner and outer rings of high attenuation, corresponding to the enhancing mucosa and *muscularis propria* and a middle ring of gray attenuation representing edema in the submucosa. Duodenal hematoma manifests as thickening of the duodenal wall with variable attenuation. Duodenal perforation is suspected when there is a retroperitoneal collection of oral contrast medium, extraluminal gas, or a lack of continuity of the duodenal wall. Fluid or hematoma in the retroperitoneum, adjacent fat stranding, and pancreatic transection can all be present in both contusion and perforation of the duodenum.

### Ulcers and duodenitis

Duodenitis refers to inflammation of the duodenum without discrete ulcer formation.

The three main causes are *H. pylori* infection, alcohol, and nonsteroidal anti-inflammatory drugs abuse.

It may manifest at UGIS and CT as duodenal wall thickening, nodules (enlarged Brunner glands), deformity of the duodenal bulb, and erosions.

Duodenal peptic ulcers are almost always caused by H. pylori infection. 5% of them occur at the duodenal bulb and are not considered a premalignant condition.

UGIS shows a round or oval pocket of barium filling the ulcer, with an edematous collar of swollen mucosa, absent mucosal pattern, no contraction with the peristalsis, and thickened folds radiating to the ulcer niche [25].

**Fig. 32:**
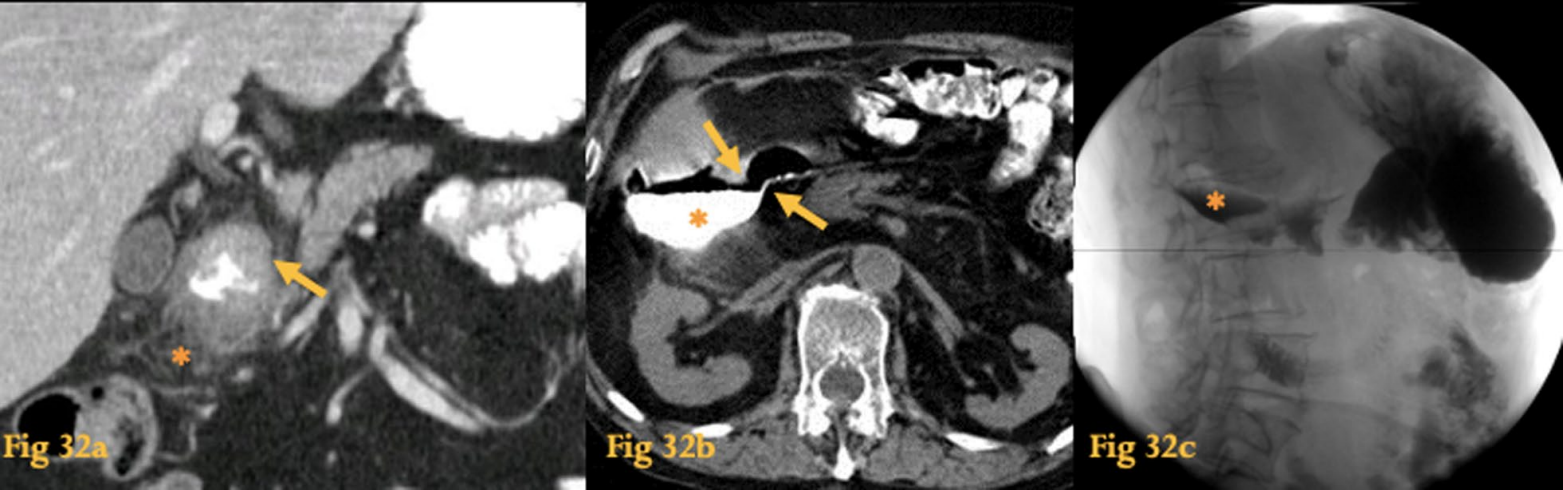
Ulcers and duodenitis. Abdominal CT of a case of duodenitis (**a**): the lumen of the duodenum is irregular; the wall is thickened (arrow) and there is adjacent fat stranding (asterisk). Plain abdominal CT of another patient (**b**) clearly shows a duodenal ulcer perforation (arrows) and accumulation of gas and oral contrast in the right anterior pararenal space (asterisk). The corresponding upper GI study performed with water-soluble contrast agent (**c**) shows retroperitoneal leakage of contrast medium (asterisk)

Complications include bleeding, perforation, and stricture, in which cases CT has better accuracy [2] (Fig. 32). Interventional radiology has a major role in the treatment of upper GI bleeding with vascular embolization.

### Vasculitis (lupus)

Systemic lupus erythematosus is a multisystem autoimmune disorder that induces necrotizing vasculitis. Lupus vasculitis at the gut is usually multifocal, transient, migratory and can occur throughout the GI tract, but is most commonly found in the small bowel.

Whenever mesenteric ischemia occurs in young patients, at unusual sites, with a tendency to affect both the small and large intestine, and is associated with genitourinary disease, the hypothesis of vasculitis must be raised.

Radiologic findings in the various types of vasculitis often overlap [41]. Knowledge of the associated clinical manifestations can suggest the specific diagnosis.

UGIS may show evidence of separation of the bowel loops, “thumbprinting,” and mucosal fold thickening.

**Fig. 33:**
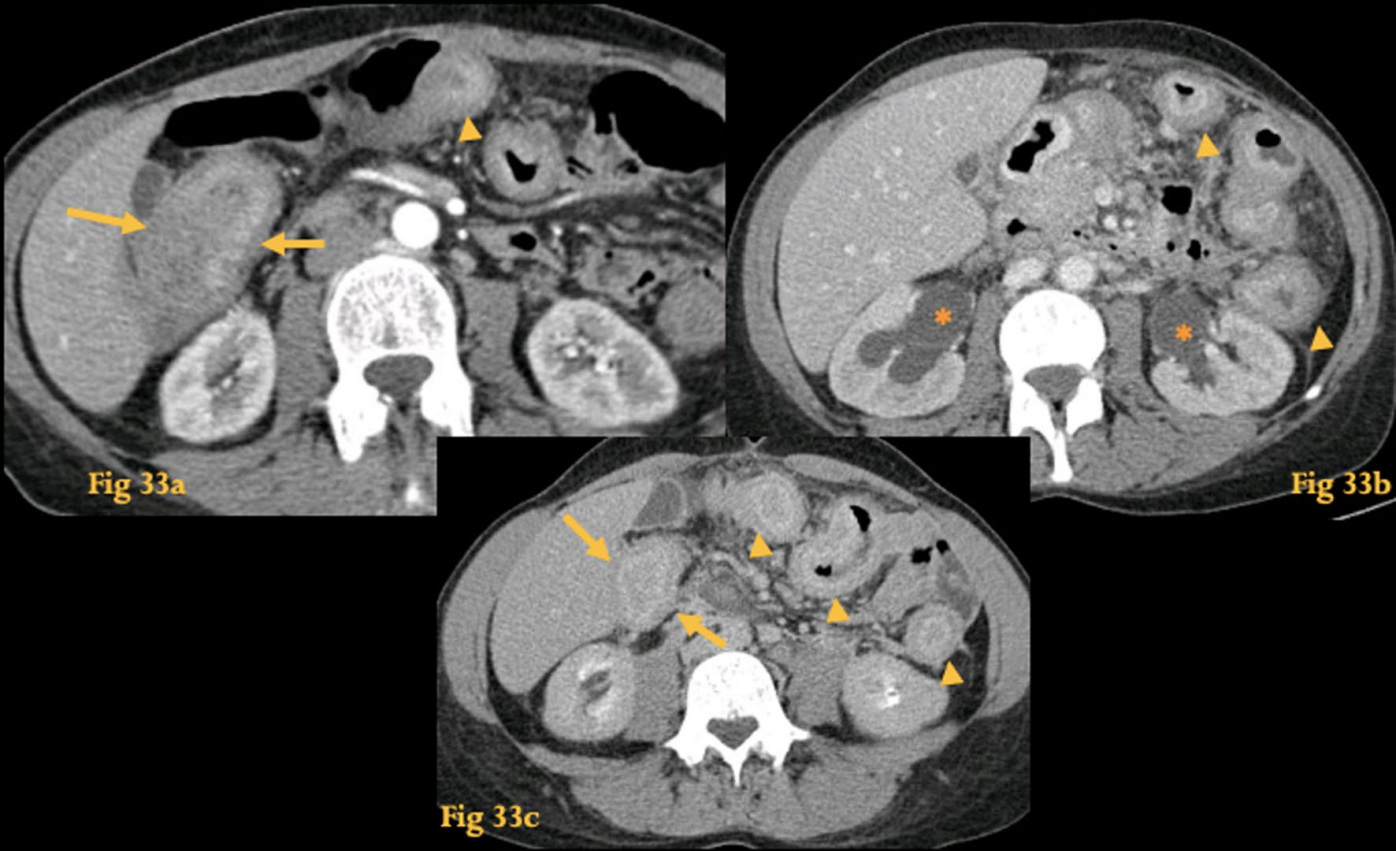
Vasculitis (lupus). Abdominal CT of a patient with systemic lupus erythematosus and antiphospholipid syndrome (**a**). There is evidence of multifocal thickening of several bowel loops with a target appearance (arrowheads), including the stomach, colon and D2 (arrows). Note also bilateral hydronephrosis (asterisks in **b**). Abdominal CT (**c)** of the same patient following 3 weeks of steroid treatment with no clinical improvement revealed resolution of some of the previous affected segments, while other bowel segments showed wall thickening for the first time

On CT inflammation of the small caliber blood vessels of the GI tract produces a variety of complications, such as intestinal ischemia/infarction, hemorrhage, ileus, ulceration, and perforation, with according imaging findings [42]. Because vasculitis may affect several vessels simultaneously, skip lesions are found and are a discriminating factor for thromboembolic mesenteric disease, which shows a continuous pattern of disease (Fig. 33). Genitourinary tract involvement (lupus nephritis, cystitis, hydronephrosis) is concurrently seen in many cases.

### (Windsock) intraluminal diverticulum

Intraluminal duodenal diverticula are rare malformations that are thought to result from chronic antegrade enteric propulsive forces on an incomplete duodenal congenital diaphragm [43]. Thus, they are composed of a double mucosal layer.

Despite being congenital, they usually manifest in adulthood in a nonspecific manner. Complications include mechanical obstruction, peptic ulcer disease, hemorrhage, cholangitis, and pancreatitis. They are more common on the descending duodenum.

**Fig. 34:**
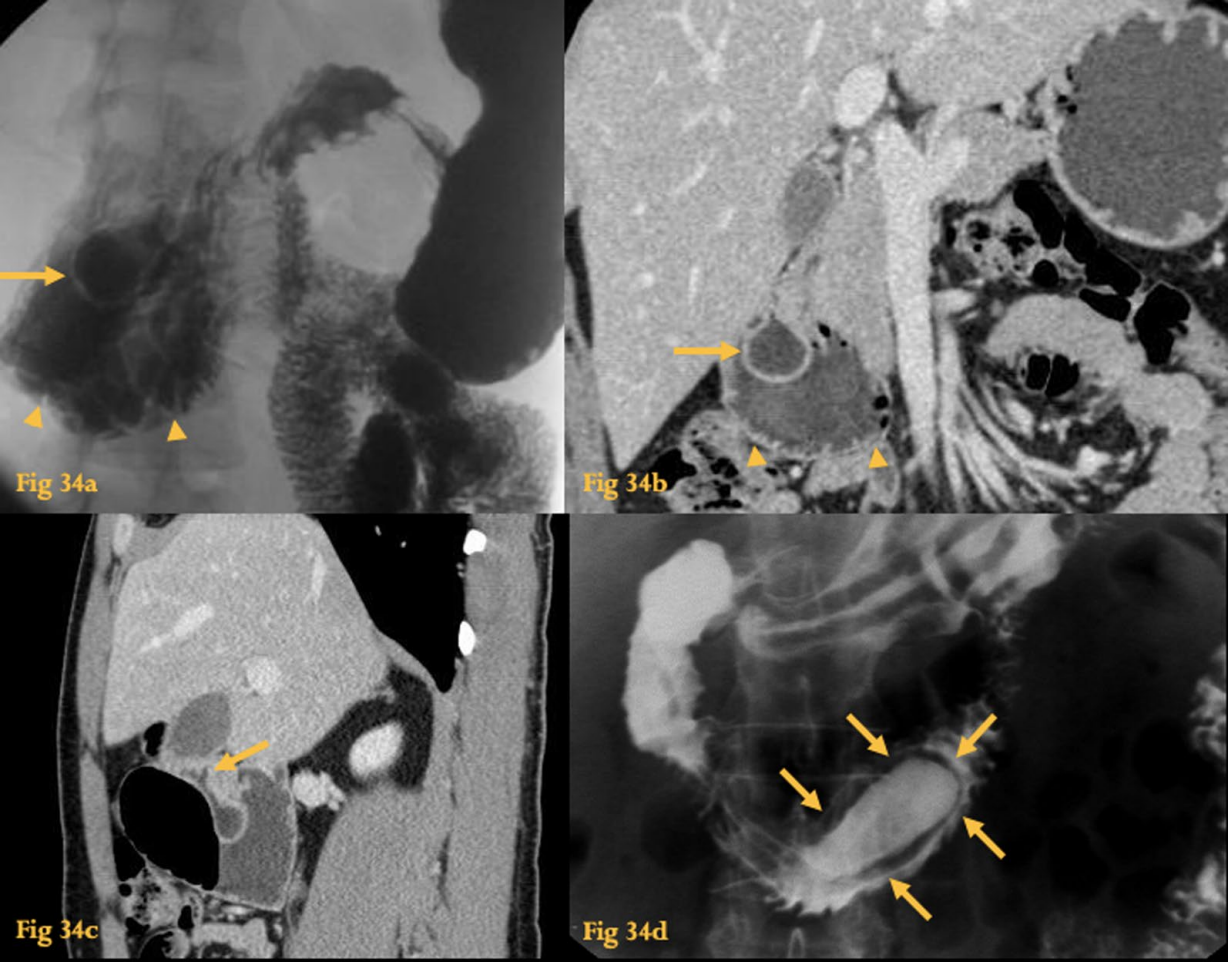
Intraluminal diverticulum. UGIS (**a**) shows a barium-filled sac surrounded by a radiolucent halo in D2 (arrow). Duodenal luminal dilatation and mucosal fold thickening are also noted (arrowheads). Abdominal CT (**b**) of the same patient with analogous findings. Sagittal reformation (**c**) clearly shows the attachment of the intraluminal diverticulum to the duodenal wall (arrowhead). UGIS of another patient (**d**) with an intraluminal diverticulum shows a radiolucent line surrounded by barium both inside and outside (arrows)

On UGIS, there is evidence of the pathognomonic “windsock sign”, a barium-filled sock within the lumen of the duodenum, whose radiolucent wall is outlined by barium both inside the diverticulum and within the true lumen of the duodenum [25] (Fig. 34a, d).

CT images show a low-density endoluminal collection in the duodenum (Fig. 34b, c). The descending duodenal caliber may be increased. Curved planar reconstruction can reveal the attachment of the diverticulum to the duodenal wall.

### Indirect signs of duodenal disease on X-ray

Duodenal diseases may produce radiographic changes in the thorax and abdomen.

**Fig. 35:**
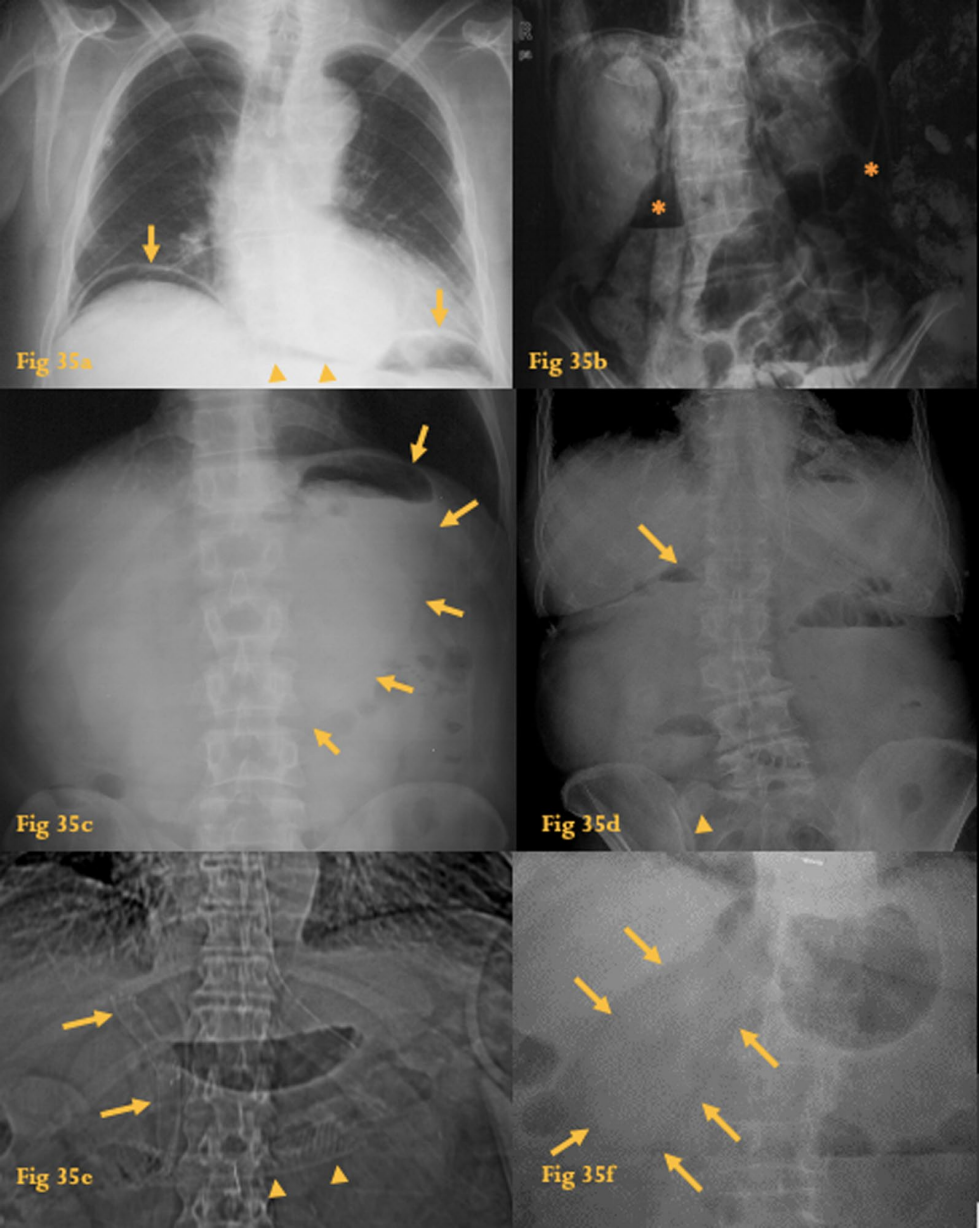
Indirect signs of duodenal disease on X-ray. Chest radiograph (**a**) shows a small amount of gas beneath the diaphragm (arrows) and the continuous diaphragm sign is also seen (arrowheads). At surgery, a perforated duodenal bulb ulcer was identified. Abdominal radiograph (**b**) shows pneumoretroperitoneum (asterisks) after duodenal rupture following ERCP. Abdominal radiograph (**c**) shows a stomach with augmented volume (arrowheads)—gastric outflow obstruction caused by multiple polyps of the duodenum in a patient with juvenile polyposis. Abdominal radiograph (**d**) shows an air-fluid level in the gallbladder (arrow) and a gallstone at the right iliac fossa (arrowhead), indicative of a gallstone ileus, because of a cholecystoduodenal fistula. Abdominal radiograph (**e**) shows a large horizontal prosthesis in the upper abdomen (arrowheads) and two smaller vertical prosthesis (arrows) in the horizontal and descending duodenum, which were a palliative treatment for an obstructive duodenal adenocarcinoma. Abdominal radiograph (**f**) shows an intraluminal soft-tissue density at D2. This was a gastric GIST prolapsing into the duodenum

In fact, an abnormal radiograph may be the initial clue to the diagnosis of several illnesses of the duodenum (Fig. 35).

### Roux-en-Y anastomosis

A Roux-en-Y enteric anastomosis was first described by surgeon Cesar Roux as a means to bypass an obstructed stomach due to recurrent ulcer attacks. Nowadays, the procedure is used as a means to re-route food or gastrointestinal secretions in other conditions such as gastric bypass (for obesity), following partial or complete gastrectomy (for gastric and oesophageal cancer), chronic pancreatitis, biliary duct obstruction (common duct strictures, choledochal cysts), etc. It comprises an anastomosis of the distal divided end of the small bowel to another organ such as the stomach or esophagus and an end-to-side anastomosis between the biliopancreatic limb and the small bowel distal to the cut end [44].

The duodenum is in its normal location and is part of the biliopancreatic limb, which measures about 100 cm in length.

An opening in the transverse colon mesentery is created for passing the Roux limb of the jejunum, so internal hernias are a possible complication of this procedure.

**Fig. 36:**
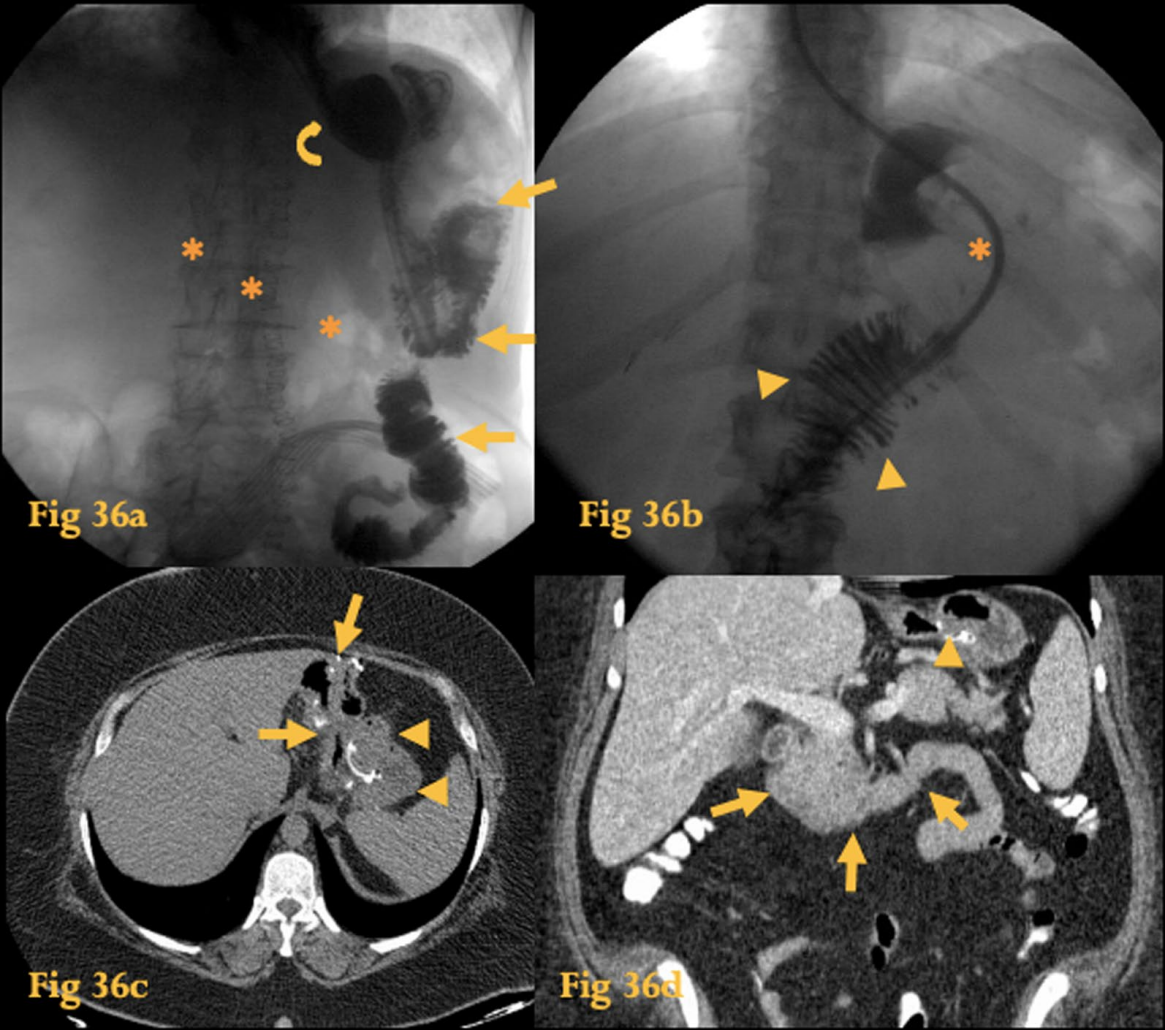
Roux-en-Y anastomosis. UGIS with water soluble contrast of a patient who underwent partial gastrectomy with Roux-en-Y anastomosis for an adenocarcinoma of the stomach (**a**): only the gastric stump (curved arrow) and gastro-jejunal loop are opacified (arrows); the biliopancreatic loop is not (asterisks in its supposed location). Fluoroscopic study with water soluble contrast of an obese woman who underwent gastric bypass with Roux-en-Y anastomosis (**b**): note the jejunal mucosal pattern (arrowheads) right after the supposed location of the gastro-jejunal anastomosis (asterisk). Abdominal CT of the same patient (**c**): the surgical clips are seen at the anastomosis between the gastric stump (arrows) and the jejunal loop (arrowheads). CT coronal reformation (**d**) shows the gastro-jejunal anastomosis (arrowhead) and the biliopancreatic limb, which includes the duodenum in its normal location (arrows)

On UGIS, only the Roux loop is opacified; the biliopancreatic limb is usually not opacified (Fig. 36a, b).

CT reflects the new anatomy, with surgical clips seen at the anastomotic sites [2] (Fig. 36c, d).

### Z-shaped duodenum (malrotation)

Malrotation is the congenital abnormal position of the bowel within the peritoneal cavity and usually involves both small and large bowel.

The term applies to a wide range of intestinal anomalies, from omphalocele in newborns to asymptomatic nonrotation of the bowel in adults, and the positions of the duodenojejunal junction and colon depend on the developmental stage the embryologic rotation failed.

It is usually diagnosed in the first year of life, with a clinical picture of bilious vomiting because of duodenal obstructive bands or midgut volvulus.

In nonrotation, an important minority and subtype of malrotation, the small bowel is located predominantly on the right side and the colon on the left side within the peritoneal cavity and complications such as volvulus are less frequent.

UGIS may depict a Z-shaped configuration of the duodenum in the presence of obstructing peritoneal bands or a corkscrew-shaped duodenum in the presence of volvulus, in newborns and young infants; the duodenojejunal junction is located lower than the duodenal bulb and to the right of the expected position.

**Fig. 37:**
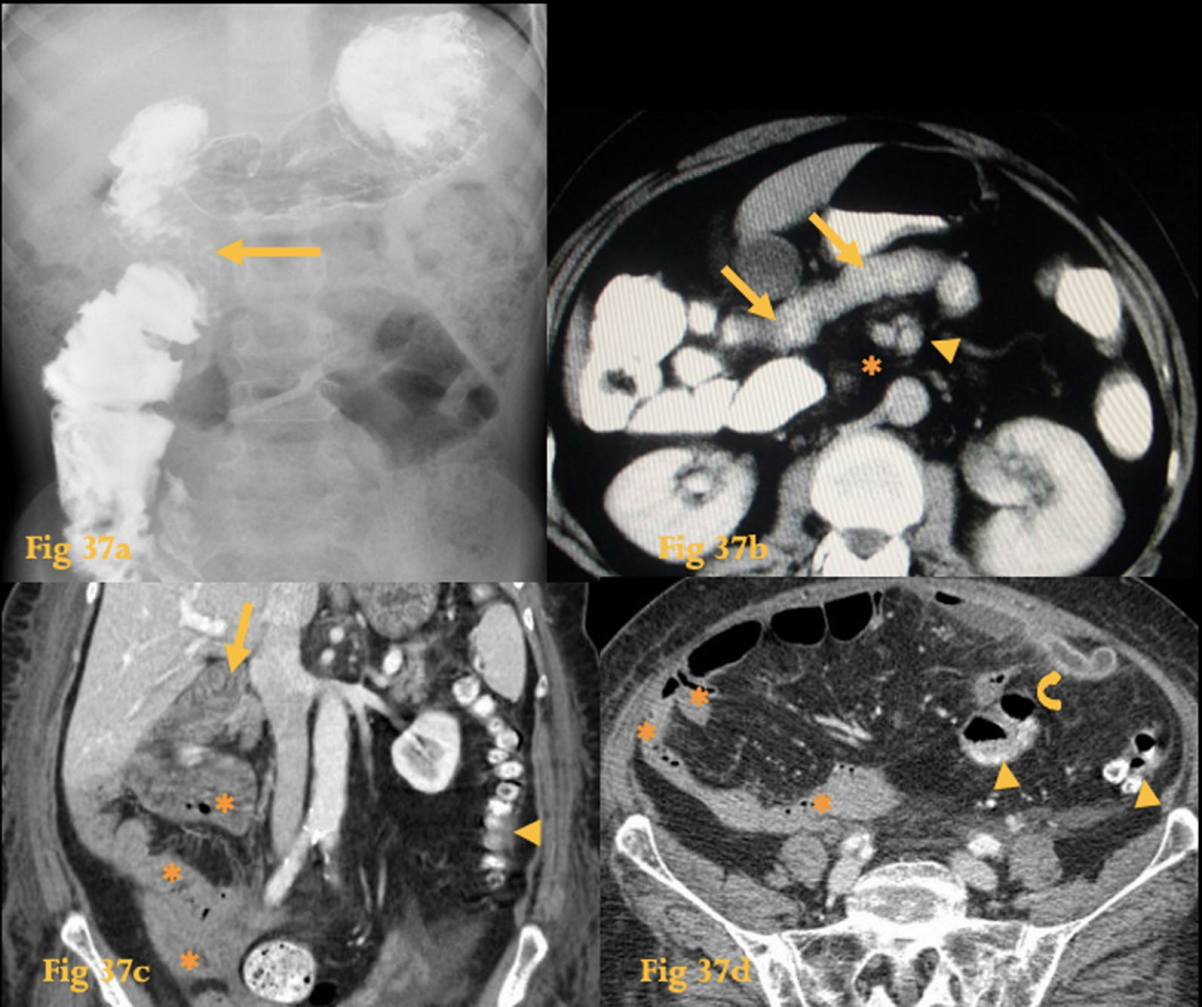
Gut malrotation. Barium study of an 8-year-old (**a**) with intestinal malrotation shows that the duodenum does not cross the midline (arrow). Abdominal CT of an adult patient (**b**) reveals absence of the 2nd part of the duodenum between the aorta and SMA (asterisks). The duodenum (arrows) crosses anterior to the SM vessels, which show an inverted relationship, with the artery located on the right and the vein on the left side (arrowhead). Incidentally, this patient also had an abnormal course of the portal vein and absence of the inferior vena cava. Abdominal CT of another adult with left flank pain and fever (**c**, **d**) shows that the duodenum does not cross the midline (arrow). The small bowel is located predominantly on the right quadrants (asterisks) while the large bowel is located on the left quadrants (arrowheads). On the left flank, an enhancing and thickened, fluid-filled tubular structure (curved arrow) is seen coming out of the cecal pole, which was located on the midline—this was a rare case of an appendicitis on the left quadrants in a patient with nonrotation

On CT, the duodenum does not cross the midline between the aorta and the SM vessels. An abnormally high position of the cecum and the SM vein to the left of the SM artery may also be seen (Fig. 37).

## Conclusion

Every diagnostic radiologist should be aware of both the most frequent and the exclusive pathology of the duodenum.

CT plays the pivotal role in the diagnostic imaging of the duodenum. Nevertheless, UGIS still have utility in some conditions and are even better than CT in precise indications (for instance windsock diverticulum, SMA syndrome, ulcers, and perforation). MRI, ultrasound, and radiographs may additionally be useful in a few other settings.

## Data Availability

Data sharing is not applicable to this article as no datasets were generated or analyzed during the current study.

## Abbreviations

CD: Crohn’s disease
CT: Computed tomography
ERCP: Endoscopic cholangiopancreatography
IV: Intravenous
MR: Magnetic resonance
MRCP: Magnetic resonance cholangiopancreatography
SM: Superior mesenteric
SMA: Superior mesenteric artery
UGIS: Upper gastrointestinal series

## References

[1] Lee CC, Ng WK, Lin KW, Lai TW, Li SM (2008). Adenocarcinoma of the duodenum. Hong Kong Med J.

[2] Lee EY, Andetta HB (2019). Computed body tomography with MRI correlation.

[3] Buck JL, Elsayed AM (1993). Ampullary tumors: radiologic-pathologic correlation. Radiographics.

[4] Barat M, Dohan A, Dautry R (2017). Mass-forming lesions of the duodenum: a pictorial review. Diagn Interv Imaging.

[5] Pannala R, Brandabur JJ, Gan SI (2011). Afferent limb syndrome and delayed GI problems after pancreaticoduodenectomy for pancreatic cancer: single-center, 14-year experience. Gastrointest Endosc.

[6] Wise SW (2000). Case 24: afferent loop syndrome. Radiology.

[7] Juan Y-H, Yu CY, Hsu HH (2011). Using multidetector-row CT for the diagnosis of afferent loop syndrome following gastroenterostomy reconstruction. Yonsei Med J.

[8] Vitale J, Boni L, Brumana N (2012). Biliary ileus. Lancet.

[9] Lassandro F, Romano S, Ragozzino A (2005). Role of helical CT in diagnosis of gallstone ileus and related conditions. AJR Am J Roentgenol.

[10] Singh AK, Shirkhoda A, Lal N, Sagar P (2003). Bouveret’s syndrome: appearance on CT and upper gastrointestinal radiography before and after stone obturation. AJR Am J Roentgenol.

[11] Patil AR, Nandikoor S, Mallarajapatna G, Shivakumar S (2017). Case 248: cystic duodenal dystrophy with groove pancreatitis. Radiology.

[12] Procacci C, Graziani R, Zamboni G (1997). Cystic dystrophy of the duodenal wall: radiologic findings. Radiology.

[13] Lee HK, Park SJ, Yi BH, Lee AL, Moon JH, Chang YW (2009). Imaging features of adult choledochal cysts: a pictorial review. Korean J Radiol.

[14] Martin RF (2014). Biliary cysts. Surg Clin N Am.

[15] Ko SY (2013). A case of a duodenal duplication cyst presenting as melena. World J Gastroenterol.

[16] Eloubeidi MA, Cohn M, Cerfolio RJ (2004). Endoscopic ultrasound-guided fine-needle aspiration in the diagnosis of foregut duplication cysts: the value of demonstrating detached ciliary tufts in cyst fluid. Cancer.

[17] Karayiannakis AJ, Bolanaki H, Courcoutsakis N (2012). Common bile duct obstruction secondary to a periampullary diverticulum. Case Rep Gastroenterol.

[18] Kim JY, Lee JM, Kim KW (2009). Ectopic pancreas: CT findings with emphasis on differentiation from small gastrointestinal stromal tumor and leiomyoma. Radiology.

[19] Silva AC, Charles JC, Kimery BD, Wood JP, Liu PT (2006). MR Cholangiopancreatography in the detection of symptomatic ectopic pancreatitis in the small-bowel mesentery. AJR Am J Roentgenol.

[20] Jayaraman MV, Mayo-Smith WW, Movson JS, Dupuy DE, Wallach MT (2001). CT of the duodenum: an overlooked segment gets its due. Radiographics.

[21] Klemen M, Ramos-Andrade Daniel C-AF (2017). EuroRad.

[22] Shen C, Chen H, Yin Y (2015). Duodenal gastrointestinal stromal tumors: clinicopathological characteristics, surgery, and long-term outcome. BMC Surg.

[23] Burkill GJC, Badran M, Al-Muderis O (2003). Malignant gastrointestinal stromal tumor: distribution, imaging features, and pattern of metastatic spread. Radiology.

[24] Sedano J, Swamy R, Jain K, Gupta S (2015). Brunner’s gland hamartoma of the duodenum. Ann R Coll Surg Engl.

[25] Brant WE, Helms CA (2018). Fundamentals of diagnostic radiology.

[26] Brosens LA (2011). Juvenile polyposis syndrome. World J Gastroenterol.

[27] Applegate KE, Goske MJ, Pierce G, Murphy D (1999). Situs revisited: imaging of the heterotaxy syndrome. Radiographics.

[28] Tadesse A, Alemu H, Silamsaw M, Gebrewold Y (2018). Kartagener’s syndrome: a case report. J Med Case Rep.

[29] Thompson WM (2005). Imaging and findings of lipomas of the gastrointestinal tract. AJR Am J Roentgenol.

[30] Kim YH, Song TJ, Ryu HS (1991). A case of primary T-cell lymphoma of the duodenum. Korean J Intern Med.

[31] Loualidi A, Spooren PFMJ, Grubben MJAL, Blomjous CEM, Goey SH (2004). Duodenal metastasis: an uncommon cause of occult small intestinal bleeding. Neth J Med.

[32] Scholz FJ, Afnan J, Behr SC (2011). CT findings in adult celiac disease. Radiographics.

[33] Takeyama N, Gokan T, Ohgiya Y (2005). CT of internal hernias. Radiographics.

[34] Mbengue A, Ndiaye A, Soko TO (2015). Closed loop obstruction: pictorial essay. Diagn Interv Imaging.

[35] Shanbhogue AKP, Fasih N, Surabhi VR (2009). A clinical and radiologic review of uncommon types and causes of pancreatitis. Radiographics.

[36] Hungerford JP, Neill Magarik MA, Hardie AD (2015). The breadth of imaging findings of groove pancreatitis. Clin Imaging.

[37] Leyendecker JR, Bloomfeld RS, DiSantis DJ (2009). MR enterography in the management of patients with crohn disease. Radiographics.

[38] Bates DDB, Wasserman M, Malek A (2017). Multidetector CT of surgically proven blunt bowel and mesenteric injury. Radiographics.

[39] Kim SH, Shin SS, Jeong YY (2009). Gastrointestinal tract perforation: MDCT findings according to the perforation sites. Korean J Radiol.

[40] Lamba R, Tanner DT, Sekhon S (2014). Multidetector CT of vascular compression syndromes in the abdomen and pelvis. Radiographics.

[41] Ha HK, Lee SH, Rha SE (2000). Radiologic features of vasculitis involving the gastrointestinal tract. Radiographics.

[42] Hokama A (2012). Endoscopic and radiographic features of gastrointestinal involvement in vasculitis. World J Gastrointest Endosc.

[43] Materne R (2001). The Duodenal Wind Sock Sign. Radiology.

[44] Ritchie WP (2000). Essentials of general surgery. Southern Med J.

